# A physics primer on photon‐counting detectors in CT: Physics, signal formation, and performance

**DOI:** 10.1002/mp.70586

**Published:** 2026-07-21

**Authors:** Guang‐Hong Chen, Ruiran Lai, Ke Li

**Affiliations:** ^1^ Department of Medical Physics University of Wisconsin‐Madison Madison Wisconsin USA; ^2^ Department of Radiology University of Wisconsin‐Madison Madison Wisconsin USA; ^3^ Department of Imaging Physics University of Texas MD Anderson Cancer Center Houston Texas USA; ^4^ Department of Interventional Radiology University of Texas MD Anderson Cancer Center Houston Texas USA

**Keywords:** CT, imaging, photon‐counting detector, x‐ray

## Abstract

Photon‐counting detector CT (PCD‐CT) is moving from laboratory development to clinical deployment. However, its threshold‐bin data arise from a tightly coupled detector–readout chain that can be difficult to understand as a whole. These data are not a direct readout of photon energy, but the outcome of a sequence of physical and electronic processes that begins with x‐ray interaction and charge creation and ends with threshold decisions applied to processed electrical pulses. Understanding this chain is essential for interpreting detector behavior, evaluating performance, and identifying the origins of spectral distortion, count loss, and instability under clinical operating conditions. This primer presents a physics‐grounded framework for semiconductor photon‐counting detectors in CT by organizing the discussion around a single causal chain: energy deposition, charge creation, charge transport, signal induction, pulse formation, and final event counting and multi‐threshold energy binning. Within this framework, we show how charge sharing, detector pixel geometry, dead time, pileup, dark current, contact‐controlled leakage, and operating conditions shape spectral response, count‐rate performance, threshold stability, and reproducibility at clinical flux. By linking detector physics, waveform formation, threshold logic, contact physics, operating conditions, and practical performance characterization within one coherent framework, this primer aims to give medical physicists and imaging researchers a scientifically rigorous and operationally useful understanding of how photon‐counting CT detectors work and what governs their performance.

## 1 INTRODUCTION

Photon‐counting detector CT (PCD‐CT) has progressed from laboratory prototypes to clinical and near‐clinical systems, with multiple vendor platforms now supporting routine patient imaging and an expanding body of technical and clinical evidence. Although current implementations differ substantially in detector pixel size, superior–inferior anatomical coverage, readout architecture (including the number of energy bins), and sensor material (e.g., CdTe, CZT, and Si), they share a common objective: to convert the post‐object polychromatic X‐ray photon stream into multi‐threshold, energy‐binned counts through a single high‐frame‐rate measurement chain. This architecture addresses several long‐standing goals in CT imaging, including improved spatial resolution, improved dose efficiency, reduced electronic‐noise penalties through low‐energy thresholding, and simultaneous spectral imaging for material‐specific image generation within clinically practical workflows.

Driven by sustained efforts from both academia and industry, multiple commercial PCD‐CT platforms have now either received regulatory approval or are approaching clinical deployment (Table 1). As a result, the field has entered a new phase in which the scientifically and technically attractive design goals of PCD‐CT can be assessed against measurable practical performance and clinical utility.

**TABLE 1:** Current photon‐counting detector (PCD) CT systems on market from different vendors.

Vendor	Sensor	Z (mm)	Δx (mm)	Energy bins	Development status
Siemens	CdTe	57.6	0.20	4	FDA Cleared
Samsung	CdTe	10.0	0.63	3	FDA Cleared
Canon	CZT	40.0	0.21	5	FDA Cleared
GE	Si	80.0	0.40	8	FDA Cleared
Neusoft	CZT	80.0	0.32	5	NMPA Cleared
United Imaging	CZT	40.0	0.21	5	NMPA Cleared

*Note*: Z denotes the collimated x‐ray beam width along the superior/inferior direction, measured at isocenter. Δx denotes the finest reconstruction slice thickness.

Abbreviations: FDA, U.S. Food and Drug Administration; NMPA, National Medical Products Administration (China).

To examine the growth of the modern PCD‐CT literature over approximately the past decade—that is, the era beginning with the installation of early research systems for extensive preclinical evaluation^1^, ^2^—we performed a title‐based PubMed search using the explicit keywords “photon‐counting CT,” “photon‐counting detector CT,” “PCCT,” and “PCD‐CT.” This search identified 777 papers published through the end of 2025.

**FIGURE 1:**
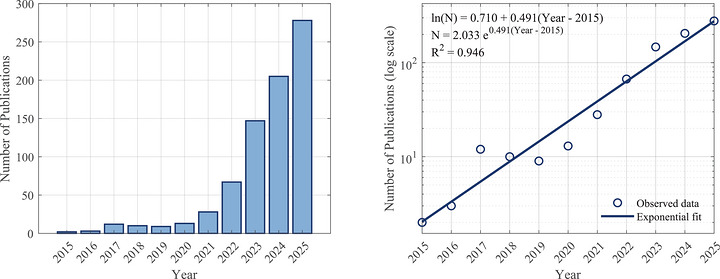
Growth of the photon‐counting CT literature over the modern development era. **Left**: annual number of title‐matched PCD‐CT publications identified by the PubMed search described in the text. **Right**: the same data on a semi‐logarithmic scale with exponential regression, showing that the recent literature expansion is approximately exponential. This growth helps motivate the need for a unified physics‐grounded framework for understanding detector operation, non‐idealities, and performance assessment.

The yearly publication histogram in Figure 1 suggests a clear multi‐stage evolution of the field. During the early period (before 2015), publication activity remained limited, consistent with an incubation phase dominated by proof‐of‐concept and laboratory studies. During the subsequent period (∼2015–2020), following the introduction of preclinical prototype systems at academic institutions such as the Mayo Clinic, the NIH Clinical Center, and the German Cancer Research Center (DKFZ), annual output increased gradually as research expanded into preclinical system performance evaluation, detector development, image reconstruction, material decomposition, and related technical areas. During the most recent period (∼2021–2025), following the emergence of the first U.S. FDA‐cleared clinical systems, publication activity increased sharply, rising from 28 papers in 2021 to 278 in 2025. On a semilogarithmic plot (Figure 1), the data are approximately linear, indicating that the literature growth through 2025 can be reasonably approximated as exponential, with an estimated doubling time of about 1.4 years. These observations show that PCD‐CT has evolved from a specialized research topic into a rapidly expanding interdisciplinary field spanning detector technology, image reconstruction, quantitative imaging, and clinical translation.

This growth has been accompanied by a large and diverse review literature. Among the identified PCD‐CT papers, more than 60 are review‐type articles intended to introduce, summarize, or contextualize the technology. Broadly, these reviews can be grouped into three complementary categories: (1) physics/engineering reviews, (2) technical–clinical bridge reviews, and (3) primarily clinical/radiology reviews. This progression mirrors the maturation of the field itself: first, the establishment of detector physics and image‐formation principles; second, the translation of these principles into deployable imaging systems, protocols, and workflows; and third, the evaluation of PCD‐CT in specific organs, diseases, and radiologic subspecialties.

The **physics/engineering** reviews, although still representing a relatively small fraction of the current review literature, focus on the scientific and technological foundations of PCD‐CT. They discuss operating principles of photon‐counting detectors, including direct conversion, energy discrimination, detector‐material selection, pulse pileup, charge sharing, electronic‐noise behavior, spatial resolution, dose efficiency, and spectral imaging capability. Their main value is to clarify what fundamentally distinguishes PCD‐CT from conventional energy‐integrating detector (EID) CT and how detector and system design shape achievable image quality and quantitative performance.^3^, ^4^, ^5^, ^6^, ^7^, ^8^, ^9^, ^10^, ^11^ However, these reviews do not present a unified end‐to‐end framework that explicitly connects the full imaging physics chain to the final multi‐bin measurement.

A much larger body of **technical–clinical bridge** reviews occupies the translational space between imaging physics and routine clinical practice. These papers typically summarize the operating principles of PCD‐CT and then interpret them in terms of practical imaging advantages, protocol design, workflow integration, dose‐reduction strategies, implementation challenges, standardization, and early multicenter experience. Many also synthesize early patient studies, compare emerging system implementations across vendors and institutions, and provide practical guidance for clinical adoption.^12^, ^13^, ^14^, ^15^, ^16^, ^17^, ^18^, ^19^, ^20^, ^21^, ^22^, ^23^, ^24^, ^25^, ^26^, ^27^, ^28^, ^29^, ^30^, ^31^, ^32^, ^33^, ^34^, ^35^, ^36^, ^37^, ^38^, ^39^ Within this category, several reviews also take important first steps toward standardization by proposing common technical terminology and acquisition protocols.

The third category comprises **primarily clinical/radiology** reviews centered on organ‐specific, disease‐specific, or specialty‐specific applications of PCD‐CT. Their purpose is to assess how the improved spatial resolution, spectral capability, artifact behavior, contrast performance, and protocol flexibility of PCD‐CT may benefit particular diagnostic tasks in cardiovascular, thoracic, abdominal, musculoskeletal, neurovascular, head‐and‐neck, pediatric, dental, and oncologic imaging.^40^, ^41^, ^42^, ^43^, ^44^, ^45^, ^46^, ^47^, ^48^, ^49^, ^50^, ^51^, ^52^, ^53^, ^54^, ^55^, ^56^, ^57^, ^58^, ^59^, ^60^, ^61^, ^62^, ^63^, ^64^, ^65^, ^66^, ^67^, ^68^, ^69^ Collectively, these reviews show that PCD‐CT is now being evaluated not only as a new CT technology, but also as a clinically relevant diagnostic imaging tool in concrete subspecialty settings.

Despite this substantial review literature, an important gap remains. As additional PCD‐CT platforms receive regulatory approval, the field increasingly requires rigorous and standardized approaches to technical assessment, commissioning, quality control, and clinical evaluation. Existing guidance documents and task‐group reports on CT performance assessment^70^, ^71^, ^72^, ^73^ provide an important foundation, but some elements may warrant re‐evaluation or targeted extension to account for characteristics such as noise behavior and artifacts arising from the distinct detector physics and readout electronics of photon‐counting systems. At the same time, ongoing clinical experience makes clear that some initially anticipated advantages of PCD‐CT are achieved only incompletely in current technical implementations, indicating that substantial opportunities remain for detector, electronics, and system‐level innovation.

These needs motivate the present primer. Rather than re‐surveying clinical studies or listing detector effects one by one, we present an end‐to‐end imaging‐physics perspective in which the measured data are traced back to their physical origin. Particular emphasis is placed on how nonidealities propagate into bin counts under clinical operating conditions. By making these links explicit, this primer aims to provide medical physicists with a coherent physics‐grounded framework for interpreting pulse‐domain and spectrum‐domain signatures, anticipating rate‐dependent and time‐dependent degradation, and relating detector behavior to CT‐specific performance metrics relevant to system evaluation, commissioning, and quality control.

## 2 FROM POST‐OBJECT PHOTONS TO THRESHOLD‐BIN DATA: A CAUSAL MEASUREMENT CHAIN

**FIGURE 2:**
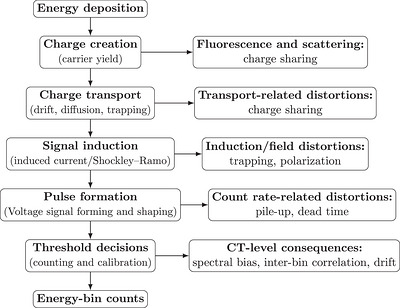
Causal measurement chain linking post‐object photons to energy‐binned digital output in a photon‐counting detector. A PCD does not measure photon energy in a single step; rather, energy‐binned counts emerge through a linked sequence of interaction physics, charge creation and transport, signal induction, pulse formation, and threshold‐based counting. The side branches indicate representative mechanisms that perturb specific stages of this chain and thereby produce CT‐relevant consequences such as spectral bias, inter‐bin correlation, and stability drift.

In x‐ray imaging, structural information of an image object along a given source‐to‐detector ray path is encoded in the post‐object x‐ray spectrum. The role of a digital detector is to compress that encoded structural information into one or more digital measurement channels. In a photon‐counting detector (PCD), this compression occurs through a causal measurement chain: x‐ray interactions deposit energy in the sensor, that energy is converted into mobile charges, charge motion induces an electrical signal, front‐end electronics shape that signal into a pulse suitable for event detection, and threshold decisions convert the pulse into discrete bin counts. Figure 2 summarizes this chain and highlights the principal links at which important non‐idealities enter before propagating into the final energy‐binned data.

This causal measurement chain makes clear that the measured “energy spectrum” in PCD‐CT is not a direct record of the incident photon‐energy spectrum. Rather, it is the outcome of a linked sequence of physical and electronic transformations applied to post‐object photons under realistic detector operating conditions. We therefore organize this primer to follow this sequence.

We begin with energy deposition through x‐ray interactions in the sensor material. These interactions convert part or all of the incident photon energy into electron‐hole pairs. The generated charge is the first electrical proxy for the post‐object photons and sets the best‐case signal scale available for subsequent processing. We then examine how these charges are transported under an applied electric field. During transport, the charge cloud can be affected by diffusion, Coulomb repulsion, trapping, detrapping, recombination, and field‐assisted carrier generation. These processes can change the spatial distribution, magnitude, and timing of the signal before it is processed by the readout electronics.

We next discuss how detector geometry helps shape the electrical signal. Pixel size, electrode design, and sensor thickness influence the time course of charge collection and electrical pulse formation, and therefore affect the stability and reproducibility needed for reliable counting. We then discuss signal induction at the collecting electrodes. In this step, electrode geometry determines the weighting field, which specifies the spatial sensitivity of the electrode to moving charge carriers and thereby reshapes the measured signal.

Finally, we examine the details of pulse formation, thresholding, counting, and binning. In these steps, the induced current is converted into a processed waveform, and its timing, amplitude, and noise properties determine whether the electronics recognize an event. Threshold‐decision logic then compresses analog pulse information into discrete energy‐bin data. At this final stage, many physically distinct upstream nonidealities appear as count loss, spectral distortion, inter‐bin correlation, spatial cross‐talk, or temporal instability.

From a medical‐physics perspective, PCD‐CT should not be understood from the final bin counts alone. One must also ask where along the imaging chain the relevant distortion first enters. For example, fluorescence originates during energy deposition and secondary‐photon transport; charge sharing arises from the spatial distribution and transport of charge carriers, together with signal induction at neighboring electrodes; pileup and dead time arise primarily during pulse formation and event handling; and calibration drift or threshold instability alters how processed pulses are converted into bin assignments. Figure 2 therefore serves not only as an outline for this primer, but also as a causal framework for interpreting detector behavior.

**FIGURE 3:**
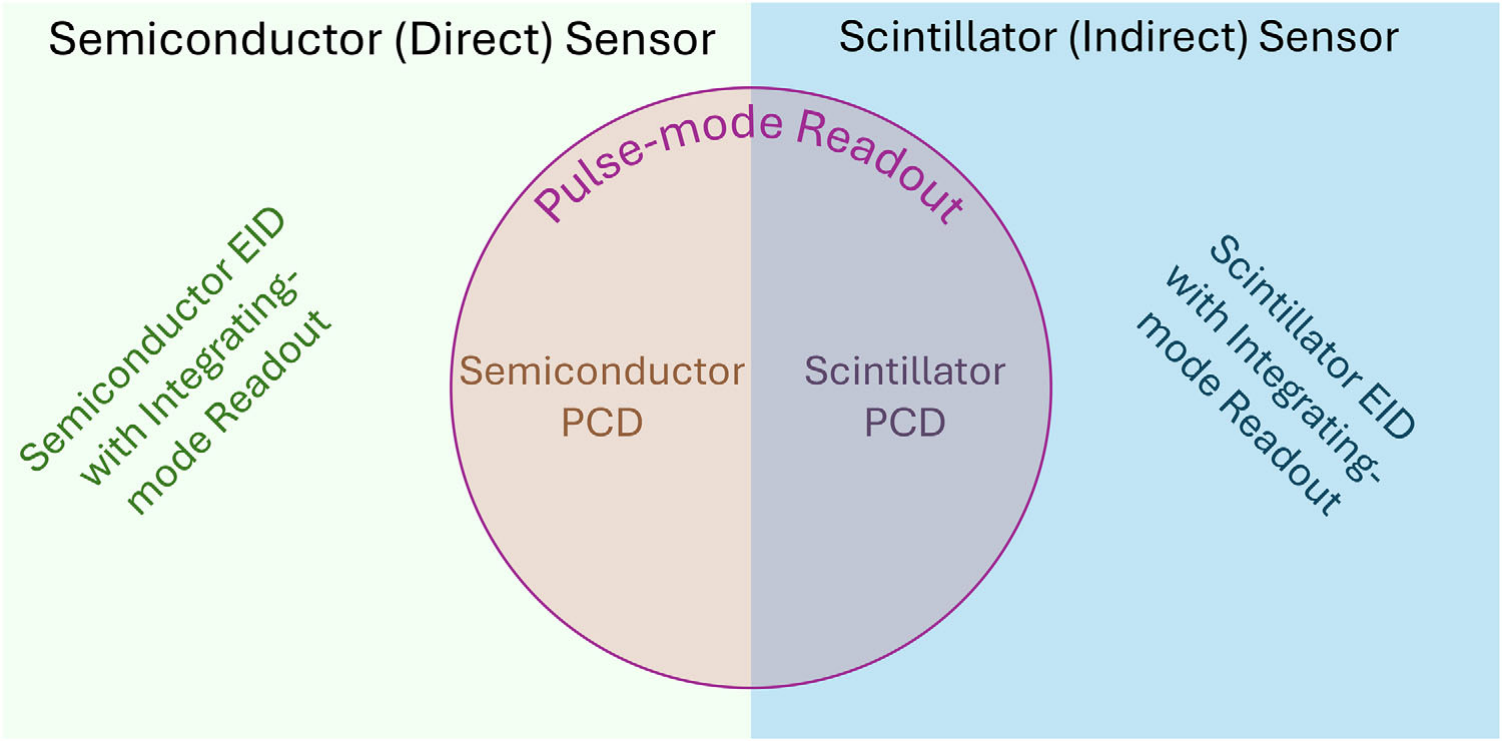
The fundamental distinction between photon‐counting detectors (PCDs) and energy‐integrating detectors (EIDs) lies in the signal readout paradigm. Most PCDs developed for CT applications employ semiconductor sensors; however, semiconductor sensing is not a fundamental requirement for photon counting, and scintillator‐based photon‐counting implementations are also under investigation for potential CT applications.

Before presenting the details of this causal measurement chain, we first define the scope of this primer. This primer focuses on semiconductor‐based PCDs, because they form the technological basis of current and emerging PCD‐CT systems. More generally, however, photon counting is not defined by the sensor material alone. The essential distinction between a PCD and an energy‐integrating detector (EID) lies in the readout paradigm: a PCD resolves individual events and applies threshold‐based counting, whereas an EID integrates signals over time. This distinction is illustrated schematically in Figure 3. Semiconductor sensors are presently the dominant choice for PCD‐CT because direct conversion produces charge carriers within the sensor material, and the transport of these charge carriers under an applied electric field allows fast event‐driven readout and high spatial resolution with small detector pixels. However, scintillator‐based photon‐counting implementations are also possible in principle and remain under active investigation.^74^, ^75^, ^76^

## 3 CHARGE CARRIER FORMATION AND TRANSPORT

The early stages of signal formation in a semiconductor x‐ray detector can be understood from two complementary perspectives. The first is a photon‐interaction perspective, which concerns how the incident x‐ray photon transfers energy to the detector material through microscopic interaction processes such as photoelectric absorption, Compton scattering, and Rayleigh scattering. The second is a semiconductor perspective, which concerns how the deposited energy is accommodated by the electronic structure of the solid (a collective physics description for all orbital electrons in the solid) and to what extent it is converted into mobile electron–hole pairs that can ultimately contribute to a measurable electrical signal.

These are not separate physical processes, but two descriptions of the same underlying physics. The photon‐interaction perspective focuses on whether, where, and by what mechanism photon energy is deposited in the detector. The semiconductor perspective focuses on how that deposited energy is converted and partitioned among electron‐hole pair generation, crystal‐lattice excitation, defect‐related carrier loss, and other internal channels that influence signal amplitude and charge‐transport behavior. Both perspectives are needed in detector physics: the probability of each microscopic photon‐interaction channel alone does not determine the overall detector response, and the charge‐generation efficiency alone does not determine whether the photon energy is captured in the sensor in the first place.

### 3.1 Energy deposition: Photon interaction perspective

**FIGURE 4:**
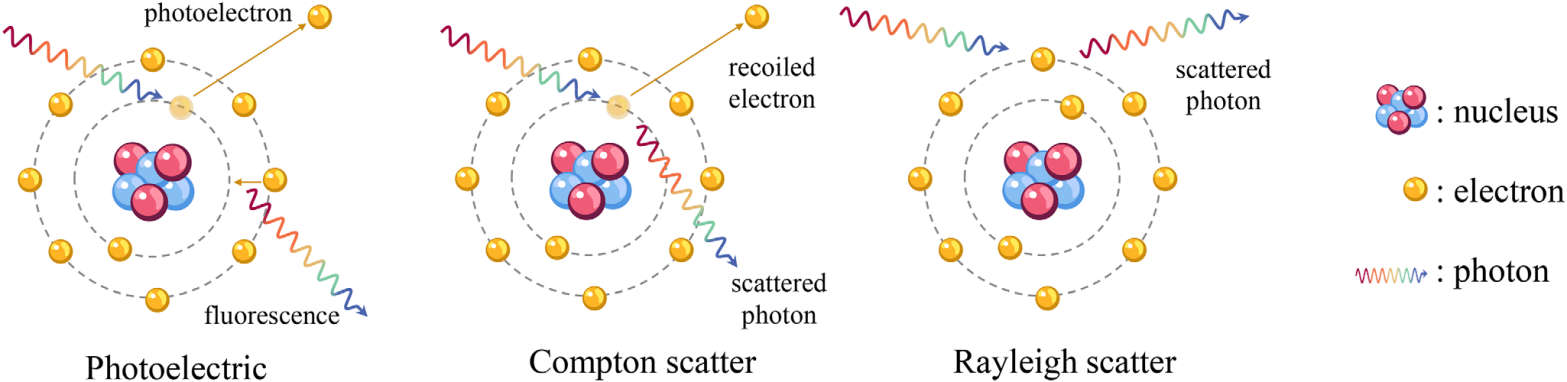
Dominant x‐ray interaction mechanisms in matter within the diagnostic energy range: photoelectric absorption, Compton scattering, and Rayleigh scattering.

When an x‐ray photon enters the sensor material, it may undergo Rayleigh scattering, Compton scattering, or photoelectric absorption (Figure 4). Through these interaction channels, the photon exchanges energy and momentum with the material, primarily through bound electrons and, to a much smaller extent, nuclei. The relative probabilities of these processes, as characterized by their corresponding cross‐sections, are governed mainly by photon energy and material composition, especially quantities related to atomic number and electron density.

In any given interaction, only part of the photon energy may be transferred locally; the remaining energy may be either retained by a scattered photon or carried away by secondary quanta (mainly orbital electrons) generated during the interaction. Energy deposition in the sensor is therefore generally not a single‐step event, but the cumulative outcome of a cascade of microscopic processes. Primary interactions produce energetic secondary electrons and, in some cases, secondary photons. These secondary quanta may undergo further interactions within the material, progressively redistributing the incident photon energy through additional scattering, excitation, and ionization events until a sort of equilibrium is established.

Among these processes in the diagnostic x‐ray energy range, Rayleigh scattering is predominantly elastic: it changes the photon direction with negligible net energy transfer to the sensor material. Its main consequence is therefore to redirect the photon trajectory, so that subsequent energy deposition may occur at a different location in the sensor. Compton scattering transfers part of the photon energy to a recoil electron while producing a scattered photon of reduced energy. That scattered photon may subsequently interact elsewhere in the sensor or escape from the material entirely. By contrast, photoelectric absorption transfers nearly all of the incident photon energy to the atom, ejecting an energetic photoelectron and leaving the atom in an excited state. Subsequent atomic relaxation may then produce characteristic fluorescence photons and/or Auger electrons. Each of these secondary quanta may in turn either deposit energy locally, migrate before interacting, or escape from the sensor.

In summary, energy transfer from an incident x‐ray photon to the sensor material is best understood as a cascade rather than as a single localized event. A primary interaction generates secondary quanta. Some of these secondary quanta deposit energy near the original interaction site, whereas others transport part of the energy to different locations or out of the material altogether. The final outcome is an overall partition of the incident photon energy into two broad components: (i) energy deposited within the sensor material, where it becomes available for subsequent charge‐carrier generation; and (ii) energy that leaves the sensor through transmitted photons or escaping secondary radiation.

#### Photon‐Interaction Considerations for Sensor Materials

The energy‐deposition perspective above provides a common physical basis for understanding semiconductor sensor‐material selection in PCD‐CT. For a given post‐object x‐ray spectrum, an ideal sensor should satisfy two closely related requirements. First, it should absorb and deposit incident photon energy with high efficiency so that the energy carried by the transmitted x‐ray beam can be maximally converted into electron‐hole pairs and, ultimately, measurable electrical signals. Second, the deposited energy from each photon should remain spatially localized. If the incident photon energy is redistributed over multiple energy‐deposition locations, the resulting electrical signals may be shared among multiple detector pixels or detector rows, leading to cross‐talk, increased counting ambiguity, and degraded energy‐bin fidelity.

In this regard, high‐Z compound semiconductors, such as CdTe and CZT, have a larger photoelectric contribution to the total attenuation coefficient in the diagnostic x‐ray energy range (Table 2). They also have high overall attenuation coefficients. These properties support compact, face‐on detector geometries with high absorption efficiency. However, photoelectric absorption may be followed by characteristic fluorescence. If a fluorescence photon escapes the original interaction site, is reabsorbed elsewhere, or leaves the sensor, both the measured energy and the apparent spatial location of the event can be distorted.^77^

**TABLE 2:** Attenuation coefficients and interaction fractions of Si, CdTe, and Cd_0.9_Zn_0.1_Te at 60 keV.

Material	Density (g/cm^3^)	${\left(\mu /\rho \right)}_{\mathrm{tot}}$ (cm^2^/g)	$\mu_{\mathrm{tot}}$ (cm^−1^)	${\left(\mu /\rho \right)}_{\mathrm{pe}}$ (cm^2^/g)	$f_{\mathrm{pe}}$ (%)	$f_{\mathrm{Compton}}$ (%)
Si	2.33	0.3206	0.747	0.1288	40.2	47.6
CdTe	6.06	6.542	39.6	6.195	94.7	1.7
Cd_0.9_Zn_0.1_Te	5.90	6.437	38.0	6.092	94.6	1.7

*Note*: The linear attenuation coefficient is calculated as $\mu_{\mathrm{tot}}=\rho {\left(\mu /\rho \right)}_{\mathrm{tot}}$. The photoelectric and Compton fractions are defined relative to the total attenuation coefficient, including Rayleigh scattering.

In contrast, silicon represents a different trade‐off. Because silicon has a lower atomic number and lower density, its attenuation coefficient is much smaller than that of CdTe or CZT in the diagnostic x‐ray energy range (Table 2). Efficient energy deposition therefore requires a long interaction path length, which motivates the edge‐on deep‐silicon geometry.^9^ In this geometry, photons traverse several centimeters of silicon along the incident‐photon direction, while the generated charge is collected over the much smaller wafer thickness.^77^, ^78^ The same low‐Z physics also makes Compton scattering a large fraction of the total interaction probability in silicon.^79^, ^80^ As a result, the main material‐induced challenge in deep‐silicon detectors is intra‐detector scatter: a Compton‐scattered photon may carry part of the incident photon energy away from the original interaction site and deposit it elsewhere, producing spatial spreading of energy deposition and cross‐talk among detector pixels or rows.^80^, ^81^ Mitigation strategies such as tungsten septa^78^ or high‐Z scintillating elements^82^, ^83^ may therefore be needed to manage silicon‐scattered photons. Tungsten septa can block scattered photons and reduce inter‐row cross‐talk, whereas scintillating elements can convert some of these scattered photons into useful signal.^81^, ^82^

### 3.2 Charge‐carrier creation: Semiconductor perspective

We now turn to the semiconductor perspective and examine how locally deposited energy in the sensor is converted into mobile charge carriers. A defining feature of a semiconductor sensor is its electronic energy band structure, which arises from the periodic atomic arrangement of the crystal lattice. At thermal equilibrium, the valence band is nearly fully occupied, whereas the conduction band is nearly empty, and the two are separated by a finite bandgap. From the semiconductor perspective, deposited x‐ray energy contributes to the measurable signal only insofar as it is converted into mobile electron‐hole pairs, with electrons excited to the conduction band and corresponding holes left in the valence band. This e‐h pair creation process sets the ideal upper bound on the total charge initially available for signal formation and establishes the initial condition for subsequent charge transport.

#### Mean charge‐carrier yield

The ideal upper bound on the available signal charge is set first by the mean number of generated electron–hole pairs, ${\bar{N}}_{eh}$, which, to leading order, scales linearly with the locally deposited energy:

(1) $${\bar{N}}_{eh}=\frac{E_{\mathrm{dep}}}{E_{i}},$$

where $E_{\mathrm{dep}}$ denotes the locally deposited energy in the sensor, and $E_{i}$ is the mean ionization energy required to create one electron‐hole pair. It is important to emphasize that $E_{i}$ is generally larger than the bandgap of the semiconductor due to the fact that not all deposited energy is converted into e‐h pair creation. Instead, a portion is dissipated through other microscopic channels, including lattice vibrations (i.e., phonon generation) and related relaxation processes.

Representative material properties of several semiconductor sensor materials relevant to the current PCDs are summarized in Table 3. Using these values, one may estimate the corresponding charge yields for a representative diagnostic‐energy deposition of $E_{\mathrm{dep}}=60\;\mathrm{keV}$, as shown in Box 3.1.

**TABLE 3:** Representative physical properties of single‐crystal Si, GaAs, CdTe, and Cd_0.9_Zn_0.1_Te at T=300K.

Material	Density (g/cm^3^)	$Z_{\mathrm{eff}}$	$\mu_{n}$ [cm^2^/(V·s)]	$\mu_{p}$ [cm^2^/(V·s)]	Bandgap (eV)	$E_{i}$ (eV)
Si	2.33	14	1350	480	1.12	3.62
CdTe	6.06	50	1100	100	1.52	4.43
Cd_0.9_Zn_0.1_Te	5.90	49	1000	90	1.57	4.64
GaAs	5.32	32	8500	400	1.44	4.25

*Note*: $\mu_{n}$ and $\mu_{p}$ denote the electron and hole mobilities, respectively, and $Z_{\mathrm{eff}}$ denotes the effective atomic number of the material.

Equation (1) further implies the best‐case charge scale initially available to the front‐end electronics:

(2) $$Q_{0}=e\;{\bar{N}}_{eh}=e\;\frac{E_{\mathrm{dep}}}{E_{i}},$$

where e is the elementary charge. This quantity provides a natural reference for subsequent discussions of pulse‐height encoding of deposited energy, before the effects of incomplete charge collection, charge sharing, pulse pile‐up, and electronic signal processing are introduced.

#### Fluctuations in charge‐carrier yield

Charge‐carrier creation is inherently stochastic. Consequently, even for fixed deposited energy $E_{\mathrm{dep}}$, the actual number of generated e‐h pairs fluctuates about its mean value ${\bar{N}}_{eh}$. These fluctuations are not, in general, Poissonian. The reason is that e‐h pair creation arises through a correlated cascade of microscopic energy‐partition processes constrained by overall energy conservation, rather than through statistically independent e‐h pair creation events. As a result, the variance of the generated electron‐hole pair number is typically smaller than the Poisson value; the pair‐generation process is therefore sub‐Poissonian. This variance is commonly written as^84^, ^85^

(3) $$\mathrm{Var}\left(N_{eh}\right)\approx F\;{\bar{N}}_{eh},$$

where F is the Fano factor of the sensor material. For semiconductor detectors, F<1, reflecting suppressed fluctuations in charge‐carrier generation relative to a Poisson process. As will be discussed later, the pulse amplitude is limited by the total charge produced in a single x‐ray interaction and is used to estimate photon energy. Therefore, fluctuations in charge generation introduce an intrinsic uncertainty in energy measurement. Equation (3) consequently represents an intrinsic contribution to the fundamental energy‐resolution limit, even before transport loss, charge sharing, and readout noise are taken into account.^86^

#### A note on sensor‐material selection in the context of the imaging chain

From the standpoint of charge‐carrier creation alone, the dominant material dependence enters through $E_{i}$, which determines the best‐case carrier yield via Equation (1). In that limited sense, silicon generates more electron–hole pairs per unit deposited energy because its $E_{i}$ is smaller. In PCD‐CT, however, as discussed before, sensor‐material selection must also account for x‐ray interaction physics. Higher‐Z and higher‐density sensors, such as CdTe and CZT, provide substantially greater x‐ray absorption efficiency at diagnostic energies, whereas lower‐Z materials, such as silicon, often require alternative design strategies, including increased detector thickness or modified detector geometry, to achieve comparable detection efficiency. Accordingly, the carrier yield described in this subsection should not be interpreted in isolation, but rather as one part of a broader detector‐performance tradeoff linking photon interaction, charge generation, charge transport, and electronic readout.

**Box 3.1: How much charge does one x‐ray interaction create?**

For a representative locally deposited energy in the diagnostic x‐ray range, $E_{\mathrm{dep}}=60\;\mathrm{keV}$, the mean number of generated electron‐hole pairs in a direct‐conversion semiconductor detector is

$${\bar{N}}_{eh}\approx \left\{\begin{array}{ccc} 1.35\times 10^{4}, & \text{CdTe}, & Q_{0}=e{\bar{N}}_{eh}\\ 1.29\times 10^{4}, & \text{CZT}, & \;=\left(1.602176634\times 10^{-19}\;C\right){\bar{N}}_{eh}\\ 1.66\times 10^{4}, & \text{Si}, & \;∼\left(2-3\right)\times 10^{-15}\;C=\left(2-3\right)fC. \end{array}\right.$$

Thus, a single diagnostic‐energy x‐ray interaction at 60keV typically produces only a femtocoulomb (fC)‐scale charge packet. This is the fundamental signal scale against which equivalent noise charge (ENC), incomplete charge collection, pulse‐height loss, and rate‐dependent distortion should be interpreted in PCD‐CT.

Alternatively, one can also use the mean carrier yield per unit deposited energy to characterize this conversion between charge and deposited energy:

$${\bar{N}}_{eh}\;\left(\text{per keV}\right)\approx \frac{10^{3}\left(\text{eV}\right)}{E_{i}\left(\text{eV}\right)},$$

which gives approximately

$${\bar{N}}_{eh}^{\mathrm{CdTe}}\approx 226\;\text{pairs/keV},\;{\bar{N}}_{eh}^{\mathrm{CZT}}\approx 216\;\text{pairs/keV},\;{\bar{N}}_{eh}^{\mathrm{Si}}\approx 278\;\text{pairs/keV}.$$

Even before transport loss, charge sharing, or electronic processing, a single x‐ray interaction generates only an extremely small charge, on the order of $10^{-15}\;C$, motivating the use of a charge‐sensitive amplifier (CSA) in the detector readout chain.

For comparison, in a conventional gadolinium oxysulfide (GOS)‐based EID, the absorbed x‐ray energy is first converted into scintillation light and then into electrical charge in the photodiode. The light yield of a representative GOS is about $5\times 10^{4}$ optical photons/MeV, or about 50 optical photons/keV of absorbed x‐ray energy, and the corresponding photodiode output is on the order of 20 electrons/keV.^87^ Thus, for a 60keV absorbed photon, one expects roughly $3\times 10^{3}$ optical photons and about $1.2\times 10^{3}$ electrons at the photodiode output, compared with the $∼10^{4}$ electron‐hole pairs generated directly in a PCD, as discussed above.

### 3.3 Charge‐cloud transport under a bias field

After an x‐ray interaction creates a charge carrier cloud in a semiconductor sensor, the generated carriers can contribute to the electrical signal through the charges they induce on the electrodes, even before they are fully collected. Nevertheless, for an ideal PCD, the charge carriers must be transported and sufficiently cleared from the sensor so that the charge cloud from one photon event does not overlap with that from a subsequent event. This charge carrier clearing process is controlled by an externally applied bias voltage, which establishes an electric field between the collecting electrodes. Under this field, electrons and holes drift toward their respective electrodes.

During charge‐carrier transport, several physical processes occur simultaneously, introducing nonidealities that limit the accuracy and precision of the measurement chain. The applied electric field drives carrier drift, whereas thermal diffusion and Coulomb self‐repulsion broaden the charge cloud laterally. Trapping and recombination can further reduce the mobile carrier population before full collection. Together, these processes determine the pulse duration, the collected charge, and the lateral charge spreading that contributes to signal sharing, or cross‐talk, between neighboring pixels.

To establish a simplified physics picture of charge‐carrier transport in the single‐photon regime, we begin with an idealized baseline model in which the internal electric field is treated as fixed and approximately uniform throughout the sensor,

$$E\left(r\right)\approx E_{\mathrm{bias}}.$$

This approximation neglects field nonuniformity near the pixel electrodes, as discussed further in Section 4, as well as field distortion caused by trapped‐charge buildup, or space charge, which can eventually lead to polarization. Nevertheless, the uniform‐field model provides a useful starting point for estimating carrier transport time scales and diffusion‐limited charge‐cloud spreading.

#### Charge carrier drift, diffusion, Coulomb expansion, and loss

Under this baseline model, each charge carrier species moves with an average drift velocity proportional to the local electric field,

$$v=\pm \mu E_{\mathrm{bias}},$$

where the plus sign applies to holes, the minus sign to electrons, and μ denotes the mobility of the corresponding carrier species, as summarized in Table 3. Superimposed on this directed drift are charge‐cloud broadening processes. Thermal diffusion causes the charge cloud to spread during transport, while Coulomb self‐repulsion among like carriers can further increase its lateral extent. In contrast, trapping and recombination reduce the number of mobile carriers before full collection and are often characterized by an effective carrier lifetime τ.

Two quantities are especially important for detector operation. The first is the charge collection time $t_{\mathrm{col}}$, which sets the characteristic duration of the induced‐current pulse. The second is the lateral extent of the charge cloud, which helps determine whether charge from a single interaction remains within one pixel or is shared among neighboring pixels. For compactness, the scaling relations below are written for a generic carrier species. They can be applied separately to electrons and holes with their respective mobilities, diffusion coefficients, and effective lifetimes.

#### Two key transport scalings

In a planar detector with electrode separation distance d and bias voltage V, the approximately uniform field E=V/d gives a characteristic collection time

(4) $$t_{\mathrm{col}}\approx \frac{\ell_{\mathrm{drift}}}{\mu E},$$

where $\ell_{\mathrm{drift}}$ is the relevant drift distance. For order‐of‐magnitude estimates, one often takes $\ell_{\mathrm{drift}}∼d$, although the actual drift distance depends on interaction depth and carrier type. During this collection time, thermal diffusion produces a lateral spread of order

(5) $$\sigma_{⊥}∼\sqrt{2D\;t_{\mathrm{col}}},$$

where D is the diffusion coefficient. Equation (4) shows that slower carriers or weaker fields increase the collection time, while Equation (5) shows that longer collection times lead to greater diffusion‐driven lateral spreading. These two simple relations provide a useful diffusion‐only baseline for connecting charge‐transport physics to the propensity for charge sharing.

It is well known that, in the nondegenerate, near‐equilibrium regime, the carrier mobility and diffusion coefficient in a semiconductor are related by the Einstein relation,^88^

$$\frac{D}{\mu }\approx \frac{k_{B}T}{q},$$

where D is the carrier diffusion coefficient, μ is the carrier mobility, $k_{B}$ is the Boltzmann constant, T is the absolute temperature, and q is the magnitude of the elementary charge. Substituting this relation into Equation (5) yields the desired diffusion‐only baseline for lateral spread. For a planar detector with E=V/d,

(6) $$\sigma_{⊥}^{2}=2D\;t_{\mathrm{col}}\approx 2\frac{k_{B}T}{q}\frac{d}{E}=2\frac{k_{B}T}{q}\frac{d^{2}}{V}.$$

An important feature of this expression is the cancellation of the mobility dependence.^89^ The charge‐collection time is inversely proportional to mobility, whereas the diffusion coefficient is proportional to mobility through the Einstein relation. As a result, the diffusion‐only estimate of charge‐cloud broadening is independent of carrier mobility.

#### Charge‐cloud expansion under Coulomb repulsion

In addition to thermal diffusion, the charge cloud may broaden through Coulomb self‐repulsion among like carriers. A simple scaling estimate can be obtained by modeling one polarity component of the charge cloud as a packet with total charge Q and characteristic radius $R_{C}\left(t\right)$. In the mobility‐limited regime, the outward expansion speed $v_{C}$ is proportional to the carrier mobility and the electric field generated by the charge packet itself,

$$v_{C}=\frac{dR_{C}}{dt}∼\mu E_{\mathrm{Coul}},$$

where $E_{\mathrm{Coul}}$ denotes the Coulomb field associated with the charge packet. Near the cloud boundary, this field scales as

$$E_{\mathrm{Coul}}∼\frac{Q}{4\pi \varepsilon R_{C}^{2}},\;\varepsilon =\varepsilon_{0}\varepsilon_{r},$$

where $\varepsilon_{0}$ is the vacuum permittivity and ε is the permittivity of the sensor material. Therefore,

$$\frac{dR_{C}}{dt}∼\frac{\mu Q}{4\pi \varepsilon R_{C}^{2}}.$$

Integrating this scaling relation over a characteristic collection time $t_{\mathrm{col}}$ gives

(7) $$R_{C}∼{\left(\frac{3\mu Qt_{\mathrm{col}}}{4\pi \varepsilon }\right)}^{1/3}.$$

This cube‐root dependence on transport time is consistent with prior detector literature,^90^ where a characteristic $t^{1/3}$ scaling for Coulomb‐driven cloud expansion has been noted.^3^, ^90^ However, the present form makes the dependence on detector‐relevant transport and material parameters explicit, which allows one to directly perform numerical estimation. It should also be noted that electrons and holes are generated together and may partially overlap during the early stage of transport. The mutual Coulomb attraction between the two carrier species can therefore reduce the net cloud expansion relative to the one‐species estimate in Equation (7). Thus, this expression should be interpreted as an upper‐end order‐of‐magnitude estimate, rather than a precise prediction of the final charge‐cloud size.

To provide a numerical sense of the relative magnitude of diffusion‐driven and Coulomb‐driven broadening, Box 3.2 gives representative estimates for the same detector setting. Taken together, these estimates suggest a characteristic lateral cloud size on the order of several tens of microns, consistent with experimentally inferred charge‐cloud dimensions from cascade‐model analyses.^91^

**Box 3.2: How fast is charge transport, and how large is charge‐cloud spreading?**

For a sensor operated at nominal field strength

$$E≃\frac{V}{d}\approx 5\times 10^{3}\;V/\mathrm{cm},$$

for instance d=2mm,V=1000V for CdTe/CZT or d=0.5mm,V=250V for Si, the full‐thickness electron transit time is

$$t_{\mathrm{drift},e}\approx \frac{d}{\mu_{e}E}∼30-60\;\mathrm{ns}\;\text{for CdTe/CZT}\;\left(\text{or}∼7\;\mathrm{ns}\;\text{for Si}\right),$$

whereas the hole transit time in CdTe/CZT is much longer because of the lower hole mobility,

$$t_{\mathrm{drift},h}\approx \frac{d}{\mu_{h}E}∼300-800\;\mathrm{ns}\;\text{for CdTe/CZT}\;\left(\text{or}∼20\;\mathrm{ns}\;\text{for Si}\right).$$

These are order‐of‐magnitude estimates. In practice, the relevant collection time is often shorter because it depends on interaction depth and carrier species. As discussed in Section 4, signal formation is governed mainly by the final drift stage of electrons, whereas cloud expansion accumulates over the full drift time.

As a diffusion‐only baseline, for T≃300K, Equation (6) gives

$$\sigma_{⊥}\approx d\sqrt{\frac{2(k_{B}T/q)}{V}}\approx 14\;\mu m\;\left(\approx 7\;\mu m\;\text{for Si}\right),$$

whereas Equation (7) gives the Coulomb‐self‐repulsion estimate

$$R_{C}\approx {\left(\frac{3\mu Qt_{\mathrm{col}}}{4\pi \varepsilon }\right)}^{1/3}\approx 31\;\mu m\;\left(\approx 18\;\mu m\;\text{for Si}\right),$$

for representative values Q∼2.5fC, $\varepsilon_{r}\approx 10.9$ for CdTe/CZT and 11.7 for Si, with $t_{\mathrm{col}}∼50\;\mathrm{ns}$ for CdTe/CZT and ∼7ns for Si.

These estimates highlight a few important points. First, electron transport is much faster than hole transport, especially in CdTe/CZT, so the early induced signal is often electron‐dominated, whereas hole motion more readily contributes to pulse tailing and shaping‐time sensitivity. Second, both diffusion and Coulomb‐driven expansion can produce lateral charge‐cloud sizes of several tens of microns by the time charge reaches the collecting electrode. Third, even at the same electric field, a thinner sensor thickness or shorter effective transport distance reduces the transport time and hence the overall cloud size.

The numerical estimates shown in Box 3.2 suggest that both diffusion‐driven broadening and Coulomb‐driven expansion can be significant in discussions of charge‐cloud spreading. However, these numerical estimates should be interpreted only at the order‐of‐magnitude level, especially because partial electron‐hole overlap during the early stage of transport reduces the net Coulomb‐driven expansion.

The charge‐transport picture developed in this subsection provides a natural starting point for understanding the subsequent links in the causal measurement chain. The charge‐collection time sets the characteristic pulse duration, which in turn affects susceptibility to shaping‐time mismatch and high‐rate waveform overlap. The lateral spreading accumulated during transport affects the probability of charge sharing among neighboring pixels, thereby influencing pixel‐level event definition, spectral cross‐talk, and the covariance structure of energy‐bin counts.

### 3.4 Charge sharing

**FIGURE 5:**
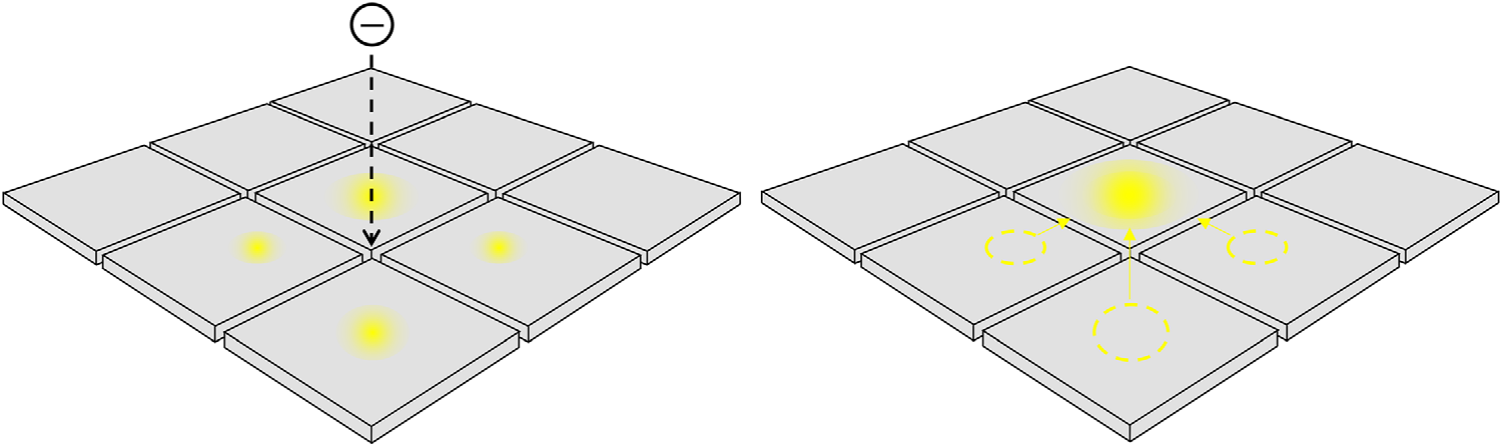
Charge sharing and coincidence summing in a pixelated photon‐counting detector. **Left**: charge sharing from a boundary‐near interaction produces signals in neighboring pixels. **Right**: coincidence summing recombines neighboring‐pixel signals from a common event, but can misassociate unrelated events at high count rates.

As discussed in the x‐ray interaction and charge‐transport subsections, an incident x‐ray photon may deposit energy at multiple spatial locations within the sensor through scattering and characteristic fluorescence reabsorption. The resulting charge clouds may further broaden during the transport process, with spatial extents readily reaching several tens of microns,^91^ comparable to detector pixel dimensions on the order of 100μm. As a result, a single photon event can induce measurable signals in multiple neighboring pixels, as illustrated in Figure 5. This phenomenon is referred to as charge sharing.

#### A simple geometric scaling for charge sharing

A simple geometric argument gives an intuitive relation between lateral cloud spreading and the likelihood of charge sharing. Consider a square pixel with pitch p, and let $\ell_{\mathrm{eff}}$ denote the effective lateral extent of the charge cloud at the end of charge transport. This effective length scale aggregates broadening contributions from both photon interaction and charge transport, without separating their individual geometric roles in the charge‐sharing model.

Charge sharing becomes likely when the center of the charge cloud lies within a boundary layer of thickness $∼\alpha \ell_{\mathrm{eff}}$ near a pixel edge. Here, α is an order‐one factor that accounts for threshold settings, electronic noise, detector geometry, including the small‐pixel effect discussed further in Section 4, and the fraction of charge that must cross a pixel boundary to trigger a neighboring pixel. If interaction locations are approximately uniform over the pixel area, then for $\alpha \ell_{\mathrm{eff}}\ll p$,

(8) $$P_{\mathrm{share}}\approx 1-{\left(1-\frac{2\alpha \ell_{\mathrm{eff}}}{p}\right)}^{2}\approx \frac{4\alpha \ell_{\mathrm{eff}}}{p}.$$

Equation (8) shows that the probability of charge sharing increases approximately linearly with the ratio $\ell_{\mathrm{eff}}/p$. Thus, charge sharing becomes more likely as the effective lateral cloud size becomes non‐negligible relative to the pixel pitch.

Although the geometric argument above provides useful intuition for the probability of charge sharing, the ultimate charge sharing behavior is also shaped by detector geometry. Pixel geometry can strongly influence the electric‐field distribution, especially in the vicinity of the electrodes (More in Section 4). Consequently, the same charge cloud may produce different multi‐pixel signal patterns in different detector designs.

Charge sharing directly affects both counting statistics and spectral fidelity. In the ideal case, a single photon interaction produces one count in one pixel with a pulse height corresponding to the deposited photon energy. With charge sharing, the same interaction may generate signals in multiple neighboring pixels, while a given pixel may receive shared charge from more than one interaction. The recorded pulse height therefore no longer corresponds uniquely to a single local photon interaction. Consequently, events may be assigned to incorrect energy bins, counted in multiple pixels, or rejected by coincidence logic, thereby distorting both the recorded counts and the measured spectrum.

#### Correction strategies

A common strategy for mitigating charge sharing is charge summing. If two or more neighboring pixels trigger within a short coincidence window, the system interprets them as originating from a common interaction and combines their measured amplitudes. In an idealized form,

(9) $${\hat{E}}_{\mathrm{sum}}\propto \sum_{i=1}^{m}A_{i},$$

where $A_{i}$ denotes the pulse‐height proxy, or more generally the charge‐related measurement, recorded in the i‐th participating pixel. In this way, charge summing can partially reconstruct the total event amplitude from spatially distributed multi‐pixel signals and thereby improve both counting accuracy and spectral fidelity. However, this correction introduces two challenges in practice:

- **Throughput penalty**: identifying coincident pixels, arbitrating among them, and combining their amplitudes increase processing complexity and may increase the effective dead time, thereby reducing counting efficiency at high flux.
- **Chance coincidences**: unrelated photons arriving within the coincidence window may be incorrectly grouped as a single event, producing artificially large summed amplitudes and distorting the measured spectrum.

Another potential charge‐sharing correction strategy is coincidence counting.^92^, ^93^ In contrast to full charge summing, coincidence‐counting methods do not attempt to reconstruct the original photon energy by explicitly summing the charge signals from neighboring pixels. Instead, they introduce one or more auxiliary coincidence counters to register temporally correlated events between adjacent pixels, thereby augmenting the ordinary energy‐bin data with information about charge‐sharing occurrence and, in multi‐energy implementations, the associated energy‐bin combinations. This approach can avoid much of the throughput penalty and architectural complexity associated with full charge summing, but it provides a less complete event‐by‐event recovery of the original photon energy.

In practice, the choice between charge‐summing and coincidence‐based charge‐sharing correction schemes must therefore be guided by the expected flux level, the spectral imaging task, and the practical constraints of detector implementation.

#### Material dependence and operating regime

Sensor material properties influence charge sharing and coincidence behavior through their effects on carrier transport, charge‐cloud spreading, trapping, and pulse formation. As summarized in Table 3, the slower hole transport in CdTe/CZT, together with transport‐induced charge trapping, can produce longer temporal tails and more depth‐dependent waveform signatures under certain operating conditions. These effects can complicate the selection of a robust coincidence window across variations in bias voltage, temperature, and sustained irradiation history. In silicon, the higher carrier mobilities and typically more uniform transport properties can lead to more consistent timing behavior, although the lower attenuation coefficient of silicon shifts detector design toward thicker or edge‐on geometries. In all cases, however, detector geometry, pixel pitch, and readout logic remain the dominant factors determining how multi‐pixel charge deposition is ultimately converted into recorded counts and energy‐bin assignments.

## 4 PULSE FORMATION AT THE ELECTRODES

Immediately after an x‐ray interaction creates electron–hole pairs in the sensor, the detector begins to respond. The electrical pulse is not generated only when carriers reach an electrode; rather, it develops continuously as electrons and holes move through the detector volume, drifting under the real electric field E(r) and diffusing due to carrier concentration gradients. In this sense, a semiconductor detector is not merely a charge‐collection device; rather, it converts carrier motion into a time‐dependent electrical waveform. Correspondingly, the detector readout does not simply register the arrival of collected charge. Instead, it measures a signal induced by carrier motion throughout the detector volume, with the observed pulse shape determined by the carrier trajectories, mobilities, trapping behavior, weighting field, and front‐end electronics.

The key question is then why carrier motion at some locations contributes strongly to the measured signal, whereas motion at other locations contributes only weakly. The answer lies in the weighting potential, a purely electrostatic quantity that describes the sensitivity of a given electrode to carrier motion at each point within the detector, as illustrated in Box 4.1. According to the Shockley–Ramo theorem,^94^, ^95^, ^96^ the charge induced on an electrode by a moving carrier is determined by the change in weighting potential along the carrier trajectory. Thus, the fraction of the final induced signal that has developed by time t depends on how much weighting potential the carrier has traversed by that time. Efficient pulse formation therefore requires the detector geometry, electric field, carrier transport, and front‐end shaping time to be matched so that a sufficiently large signal is induced and processed before subsequent photon interactions cause significant temporal overlap or pileup.

**Box 4.1: How does moving charge induce signal?**

For an electrode of interest, the weighting potential $\phi_{w}\left(r\right)$ is defined by an auxiliary electrostatic problem: set that electrode to unit potential, ground all others, remove all space charge, and solve

$$\nabla^{2}\phi_{w}\left(r\right)=0$$

in the detector volume. The corresponding weighting field is

$$E_{w}\left(r\right)=-\nabla \phi_{w}\left(r\right).$$

For a point charge q at position r(t) with velocity v(t), the Shockley–Ramo theorem^94^, ^95^, ^96^ gives the induced current on the electrode:

$$i\left(t\right)=q\;v\left(t\right)\cdot E_{w}\;\left(r(t)\right),$$

and the induced charge accumulated from $t_{0}$ to t:

$$\Delta Q\left(t\right)=-q\left[\phi_{w}\;\left(r(t)\right)-\phi_{w}\;\left(r(t_{0})\right)\right].$$

Signal formation therefore depends not only on carrier motion, but also on the weighting field and weighting potential set by the electrode geometry.

For an ideal parallel‐plate detector with cathode at z=0 and anode at z=d, the anode weighting potential is

$$\phi_{w}\left(z\right)=\frac{z}{d}.$$

In this planar limit, the induced signal varies linearly with depth. This simple geometry provides the reference case for understanding strip and pixel detectors, where the weighting field becomes strongly nonuniform.

For planar electrodes, the weighting sensitivity is broadly distributed across the detector volume. In small‐pixel geometries, such as those used in modern PCD‐CT systems, the weighting potential of each collecting pixel is strongly localized near the anode. This localization fundamentally changes pulse formation. In large‐pixel or planar geometries, the motion of both electrons and holes over much of the detector thickness can contribute appreciably to the measured signal. In small‐pixel geometries, by contrast, the measured pulse is dominated by carrier motion near the collecting anode. This also leads to approximately unipolar charge sensing in electron‐collecting detectors, because the direct contribution from hole motion is strongly downweighted, leaving electron motion near the anode as the dominant contributor to the measured signal.

For readers interested in the formal mathematical framework underlying this geometric picture, Box 4.1 summarizes the Shockley–Ramo theorem ^94^, ^95^, ^96^ and its simplest parallel‐plate limit. In the following subsection, we discuss in more detail how small‐pixel geometries influence induced signal formation at the anode.

### 4.1 Why geometry matters

The central message of the Shockley–Ramo weighting‐potential framework is that electrode geometry determines where carrier motion contributes most strongly to the readout signal. In a planar parallel‐plate detector, the weighting potential varies gradually across the detector volume from cathode to anode, so the electrode remains sensitive to carrier motion over a broad region of the detector. Signal formation is therefore distributed over much of the carrier transport path.

Surprisingly, this picture changes qualitatively when the collecting electrode becomes small relative to the detector thickness. In strip and pixel geometries, the weighting potential remains nearly flat throughout most of the detector bulk and then rises sharply only near the collecting electrode. Equivalently, the weighting field becomes strongly localized near the anode. In other words, although the detector still responds to carrier motion throughout the detector volume, it responds much more strongly to motion in the near‐anode region than to motion in the bulk. This behavior is the essence of the small‐pixel effect and one of the key reasons why pixelated semiconductor detectors behave so differently from ideal parallel‐plate detectors.^97^, ^98^, ^99^

**FIGURE 6:**
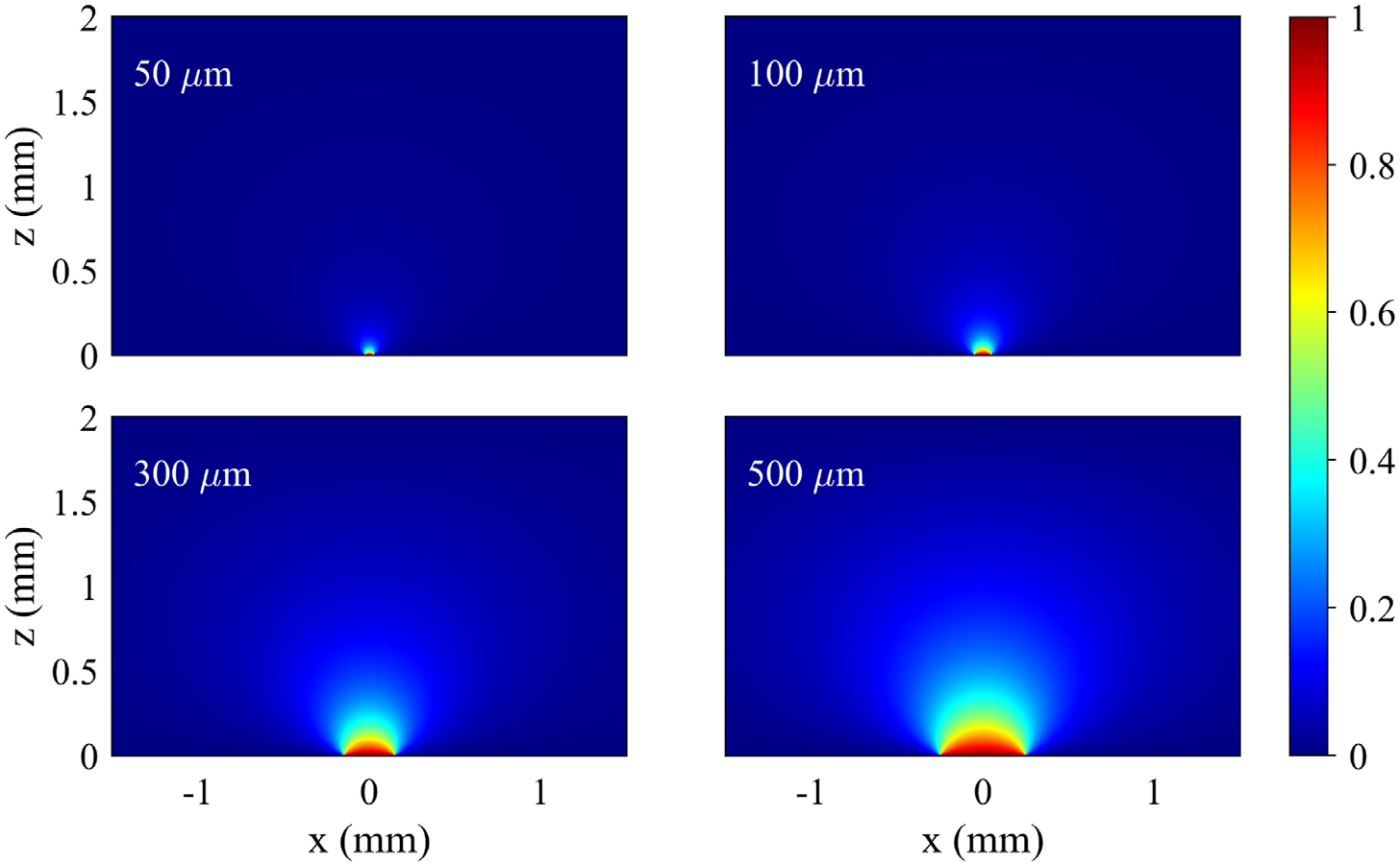
Weighting potential distribution $\Psi_{w}\left(x,z\right)$ in a strip geometry (d=2mm) for four strip widths: 50,100,300, and 500μm. As the strip width decreases, the weighting potential becomes increasingly localized near the collecting electrode, illustrating the small‐pixel effect in detector physics.

Figure 6 makes this transition visually explicit. As the detector width decreases, the weighting potential becomes increasingly compressed near the anode. Shrinking the collecting electrode therefore does not merely change the electrode size; it reshapes the sensitivity of detector to carrier motion throughout the volume.

This geometric picture also clarifies why small‐pixel detectors can behave similarly to Frisch‐grid devices.^100^ Because the weighting field is concentrated near the anode, the measured signal is dominated by electron motion close to the collecting electrode, while the direct contribution from slow hole motion is strongly reduced. The result is a more electron‐dominated and nearly unipolar pulse, achieved without the need for a physical shielding grid.^101^, ^102^, ^103^, ^104^, ^105^

### 4.2 Why small pixels reduce depth dependence

A particularly important consequence of weighting‐potential localization is the reduced depth dependence of the measured pulse. In a planar detector, the signal amplitude depends strongly on the interaction depth because the weighting potential varies steadily with depth. In a small‐pixel detector, by contrast, the weighting potential remains nearly flat throughout most of the bulk. Interactions occurring at different depths therefore experience similarly low signal sensitivity until the electrons approach the anode.

**FIGURE 7:**
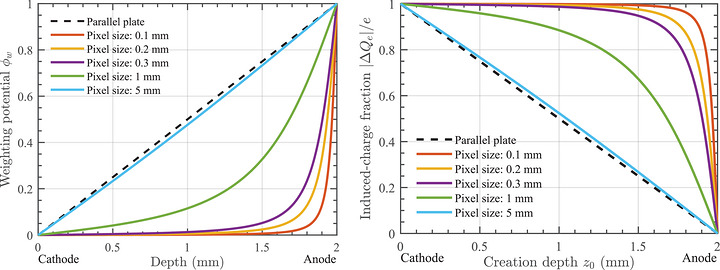
Quantitative consequences of weighting‐potential localization. **Left**: Weighting potential along the symmetry axis for a planar parallel‐plate detector and for representative small‐pixel geometries with different aspect ratios p/d. **Right**: Corresponding induced‐charge fraction for an electron drifting from creation depth $z_{0}$ to the anode. The planar case exhibits strong depth dependence, whereas the small‐pixel case becomes nearly depth invariant over much of the detector bulk.

This effect is shown quantitatively in Figure 7. For the planar geometry, the weighting potential varies linearly with depth, whereas in the small‐pixel cases it remains near zero through most of the detector thickness and rises sharply only in the near‐anode region. As a result, the induced‐charge fraction becomes much less dependent on interaction depth as the pixel size decreases relative to the detector thickness. In practical terms, small‐pixel geometries reduce a major source of pulse‐height loss and pulse‐width broadening associated with interaction depth (see the discussion of ballistic deficit in Section 5.3 and Box 5.2) and produce more sharply peaked current signals for subsequent processing and counting.

A compact way to summarize this transition is through the aspect ratio p/d, where p is the pixel pitch or strip width and d is the detector thickness. As p/d decreases, the weighting field becomes increasingly localized near the anode. In this sense, p/d provides a useful dimensionless measure of how strongly pulse formation departs from the planar limit.

### 4.3 How small pixels reshape the pulse

The importance of the weighting potential extends beyond pulse amplitude. It also reshapes the time development of the pulse itself. In a planar detector, electron and hole motion can both contribute appreciably over extended portions of the transport path. In a small‐pixel detector, however, much of the measured pulse is generated only when electrons enter the near‐anode region where the weighting field is strongest.

**FIGURE 8:**
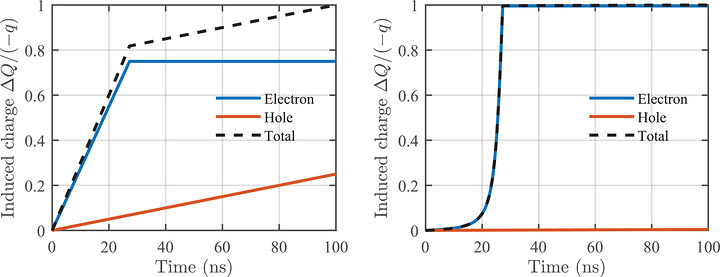
Normalized induced charge on the anode, ΔQ/q, versus time for an interaction at $z_{0}=0.5\;\mathrm{mm}$ in a CdTe detector. Electron, hole, and total contributions are shown for a parallel‐plate detector (left) and for a small‐pixel anode (right; pixel size 0.3mm). The small‐pixel geometry produces a more rapid electron‐dominated pulse and a strongly reduced direct hole contribution.

Figure 8 illustrates this time‐domain consequence directly. In the parallel‐plate case, the induced charge develops more gradually and includes a substantial hole contribution. In the small‐pixel case, the pulse rises more abruptly and is dominated by the electron component, whereas the direct hole contribution is strongly suppressed. This reorganization of signal formation is the practical signature of weighting‐field localization.

Several practical consequences follow. First, the effective signal‐formation interval can be substantially shorter than the full carrier transport time because the dominant measured signal is concentrated near the end of electron collection. Second, the reduced hole contribution mitigates hole‐induced tailing and reduces depth‐dependent pulse‐height variation, especially in sensor materials where hole transport is slow or strongly affected by trapping. Third, because the measured signal is concentrated near the anode, any mechanism that delays or degrades electron transport into that region, such as field nonuniformity, trapping, or space‐charge buildup, can still produce pulse‐height loss and rise‐time broadening. Electrode geometry therefore does not eliminate transport physics; rather, it determines which part of the transport history is weighted most strongly in the measured pulse.

This provides a natural bridge to the next section. Once the electrode geometry has shaped the analog pulse, the front‐end electronics must process that pulse for counting, thresholding, and energy discrimination.

## 5 PULSE PROCESSING FOR COUNTING

The signal induced at the electrode is not yet a count. It is an analog waveform generated by carrier motion in the sensor. To register a stream of x‐ray photons with different deposited energies, the detector must convert the signal from each photon interaction into an electrical pulse that can be evaluated rapidly and reproducibly under the high‐flux conditions encountered in diagnostic CT. This conversion is performed by the front‐end electronics. The induced current from the sensor is first integrated by the charge‐sensitive amplifier, producing a voltage signal whose amplitude is proportional to the input charge. The shaping stage then filters this voltage waveform, typically using a combination of high‐pass and low‐pass filtering, to produce a finite‐width pulse with improved pulse‐height estimation, timing behavior, and baseline stability. The processed pulse is finally compared with one or more energy thresholds to generate digital counts and energy‐bin assignments.

This step is necessary because the electronics do not act on deposited energy directly. They act on a time‐dependent electrical waveform whose amplitude and timing still reflect charge transport, weighting‐field geometry, electronic noise, and pulse overlap. Pulse processing therefore serves not only to amplify the signal, but also to produce a stable pulse‐height proxy and reliable threshold‐crossing behavior at CT‐relevant count rates.

**FIGURE 9:**
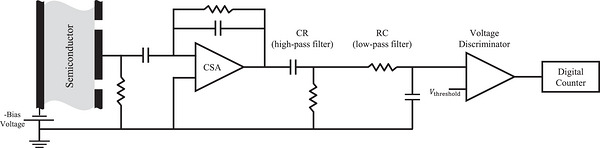
Front‐end signal chain for photon counting: sensor and electrodes → charge‐sensitive amplifier (CSA) → shaping filter → discriminator/comparator → digital counters. The chain converts a sensor‐generated analog waveform into decision quantities suitable for counting and energy binning.

Figure 9 illustrates the major functional blocks that connect sensor physics to digital counting. Although detailed implementations differ across vendors, the functional sequence is broadly similar. The sensor and electrode geometry determine the induced‐current waveform. The charge‐sensitive amplifier integrates this current into a voltage signal. Shaping filters suppress noise, control pulse width, and improve pulse‐height and timing reproducibility, as summarized in Box 5.1. Comparators then apply one or more thresholds, and digital counters record the accepted events. In this functional sense, the front end determines which features of the analog waveform ultimately govern counting and energy discrimination.

**Box 5.1: How does induced current become a decision‐ready pulse?**

The quantity that links the sensor to the front‐end electronics is the time integral of the induced current,

$$Q_{\mathrm{in}}\left(t\right)=\int_{-\infty }^{t}i\left(t^{\prime }\right)\;dt^{\prime },$$

which represents the input charge delivered to the CSA. In an idealized CSA,

$$V_{\mathrm{CSA}}\left(t\right)\approx \frac{Q_{\mathrm{in}}\left(t\right)}{C_{f}},$$

so the feedback capacitance $C_{f}$ sets the basic charge‐to‐voltage conversion. After baseline restoration, the CSA output is shaped to suppress noise and to produce a more reproducible pulse form. A common model is CR–(RC)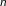 shaping with transfer function

$$H\left(s\right)=\frac{s\tau_{d}}{1+s\tau_{d}}{\left(\frac{1}{1+s\tau_{i}}\right)}^{n},$$

where $\tau_{d}$ and $\tau_{i}$ are the differentiating and integrating time constants. The key point is that pulse processing is not a passive readout step: it determines pulse width, peaking behavior, effective noise bandwidth, and the amplitude proxy that will later be compared with thresholds. Real CSAs also require reset or baseline‐restoration mechanisms.

The actual application‐specific integrated circuit (ASIC) can be far more complex than the block diagram suggests. At the schematic level, it is natural to associate counting primarily with the comparator, because the comparator is the element that makes the threshold decision. However, that decision has physical meaning only if the waveform arriving at the comparator is reproducible. The same nominal threshold corresponds to the same class of physical events only when the upstream circuitry has adequately shaped, stabilized, and equalized the pulse.^106^, ^107^, ^108^ Baseline drift provides a clear example. If leakage current or time‐varying dark current shifts the front‐end baseline, then an unchanged comparator setting no longer corresponds to the same energy threshold. Leakage‐current compensation and baseline‐restoration circuits are therefore essential parts of practical photon‐counting ASIC design.^109^, ^110^, ^111^

Much of the engineering challenge lies in making this full chain operate reproducibly at high count rate, low noise, and stable threshold settings. The comparator is the formal decision element, but reliable threshold‐based counting depends on the behavior of the entire front‐end chain.

### 5.1 Why the raw waveform is not yet a count

The current induced by the sensor is generally too small, too brief, and too variable to support reliable photon counting in its raw form. Its amplitude is associated with femtocoulomb‐scale charge packets, as shown in Box 3.1. Because its temporal structure depends on charge transport and weighting‐field geometry, its interpretation is further complicated by electronic noise, stochastic arrival times, and possible waveform overlap at high count rates. A PCD therefore cannot simply digitize the raw induced current and declare a count. It must first transform that waveform into a more reproducible decision variable.

That transformation must satisfy several requirements simultaneously. First, it must provide sufficient gain so that small sensor signals can be evaluated against stable thresholds. Second, it must suppress noise strongly enough that threshold crossings correspond predominantly to physical events rather than electronic noise fluctuations. Third, it must preserve enough temporal structure to support high‐rate operation without excessive pulse overlap, baseline drift, or ambiguous event timing. The front end is therefore designed not only to amplify, but also to regularize the waveform so that both amplitude and timing can be interpreted consistently from event to event.

At this stage, several distinct sources of amplitude bias also begin to merge into the same observable. The measured pulse height can be reduced because mobile charge is lost during transport, because the weighting‐field geometry makes some carrier motion contribute less strongly to the induced signal, or because the readout chain samples the waveform over a finite interval and therefore underweights slow components. These mechanisms arise from different physical origins, but they act on the same processed pulse that will later be compared against thresholds.

### 5.2 How the front end builds a decision‐ready waveform

The first step in the pulse‐processing chain is charge‐sensitive amplification (Figure 9). The role of the CSA is to convert the induced sensor current into a voltage whose scale is set primarily by the feedback capacitance. In doing so, it transforms the distributed charge‐motion signal discussed in the previous section into a more accessible voltage‐domain waveform for subsequent processing. The CSA therefore establishes the first usable link between sensor physics and counting electronics.

For readers interested in the compact formal framework behind this stage, Box 5.1 summarizes the basic charge‐to‐voltage and shaping relations commonly used in front‐end modeling.

The raw CSA waveform is rarely ideal for photon counting by itself. Its decay may be too long, its high‐frequency noise content too strong, and its pulse‐to‐pulse timing too irregular for robust threshold comparison. Shaping is therefore introduced to make the waveform more usable. In practice, the shaper performs three closely related functions: it suppresses wideband electronic noise, produces a controlled pulse width and peaking behavior, and accelerates effective baseline recovery so that the next event can be evaluated against a stable reference level.

The result of this processing is not a direct measurement of deposited energy yet, but a more reproducible voltage‐domain representation of the event. In other words, the front end constructs an amplitude proxy from the original induced‐current waveform. Ideally, that proxy is monotonic with deposited energy. In practice, however, it remains sensitive both to how the signal was formed in the sensor and to how the electronics process that signal in time.

### 5.3 Why shaping introduces trade‐offs

Shaping improves decision stability, but not without cost. The same filtering that suppresses noise also broadens pulses in time. Longer shaping generally reduces output noise and stabilizes amplitude estimation, but it also increases pulse width and therefore raises the probability of pileup and overlap at high count rate. Shorter shaping improves throughput, but it accepts more noise and makes the measured amplitude more sensitive to waveform details. Pulse processing is therefore governed by a fundamental trade‐off between noise suppression and rate capability.

A second trade‐off is more subtle but equally important. The shaper does not evaluate the waveform over an infinite time interval. Instead, it weights different temporal portions of the pulse according to its own transfer characteristics. As a result, a slow waveform can produce a smaller peak even when the total induced charge is similar. This effect is known as ballistic deficit. It is one of the clearest examples of how detector physics and electronics interact: the sensor may still generate nearly the same total induced charge, yet the front end can return a smaller amplitude because that charge arrived too slowly relative to the shaping time. For readers interested in the compact mathematical representation of this effect, Box 5.2 summarizes the corresponding waveform model.

This distinction is especially important in high‐Z semiconductor sensors such as CdTe and CZT, where trapping (temporary capture of charge carriers by defects in the material), polarization‐induced field reduction (a gradual weakening and distortion of the internal electric field caused by trapped‐charge buildup), and slow hole transport can introduce long temporal tails under some operating conditions. In such cases, shaping does not only clean up the pulse, it also emphasizes or suppresses particular portions of the transport history. Silicon often provides faster and more uniform transport, which can stabilize threshold decisions, but the same underlying trade‐off remains: front‐end processing does not replace detector physics. It either preserves or accentuates what the sensor delivers.

**Box 5.2: Ballistic deficit: when slow charge looks smaller**

A useful general representation of the processed waveform is the convolution

$$V\left(t\right)=\left(i*h\right)\left(t\right)=\int_{-\infty }^{+\infty }i\left(t^{\prime }\right)\;h\left(t-t^{\prime }\right)\;dt^{\prime },$$

where i(t) is the induced current and h(t) is the impulse response of the CSA and shaper chain. A convenient amplitude proxy is then

$$A\equiv \max_{t}V\left(t\right).$$

If the induced current is fast compared with the shaping timescale, then A scales nearly linearly with the input‐charge scale set by the CSA. If the current is broadened in time, however, the same total induced charge can produce a smaller peak for fixed shaping. This is ballistic deficit. The key consequence is that transport degradation can bias the measured amplitude downward even when total charge loss is modest. Ballistic deficit is therefore distinct from charge loss: nearly the same total induced charge may still produce a smaller measured pulse if the waveform is slower.

### 5.4 How high count rate changes the rules

At CT‐relevant fluence rates (∼100Mcps, where Mcps denotes million counts per second), pulse processing is limited not only by the isolated single‐event waveform quality but also by the temporal density of photon interactions. Once pulses begin to overlap, baseline stability, reset behavior, and timing logic become first‐order determinants of detector performance. Because energy thresholds are defined relative to the front‐end baseline, reproducible energy discrimination requires that the baseline remain stable across changes in count‐rate history. If the baseline shifts, recovers too slowly, or varies from event to event, the same physical signal can be assigned to different energy bins depending on the preceding events, thereby introducing count‐rate‐dependent measurement bias.

For this reason, baseline control is not a minor implementation detail. To support the high count‐rate measurements required in PCD‐CT, many front‐end architectures incorporate explicit reset or measure‐then‐reset logic so that the processed waveform is evaluated within a defined decision window and then returned to a known baseline before the next event. Such strategies can make the baseline more deterministic and help preserve the meaning of the threshold at high rates, but they also introduce additional constraints through a finite timing budget. An event may be missed if it arrives during recovery, if waveform overlap obscures the relevant rising‐edge crossing, or if a slow waveform fails to satisfy the decision rule within the allowed time window. These mechanisms directly contribute to count loss and pulse‐pileup effects that are central to PCD performance.

The same finite‐window logic can also bias energy assignment. When energy discrimination is based on threshold crossings or peak formation within a limited time window, slower pulses are preferentially shifted toward smaller measured amplitudes or lower bins. At high count rates, pulse processing therefore becomes a coupled problem involving analog filtering, event timing, and digital decision logic. Count losses and energy bin misclassification are then no longer solely determined by charge transport or by electronic noise, which sets the noise‐rejection threshold, but can arise from their interaction.

Once the front end has produced a decision‐ready waveform, the next question is how comparators and multi‐threshold logic convert that waveform into one or more registered counts at each detector pixel. From a system perspective, the central function of a PCD is to map the continuous post‐object x‐ray energy spectrum into a finite number of energy bins while preserving task‐relevant spectral information. Although the comparator performs the final threshold decision, physically meaningful counting begins upstream, when the analog waveform is shaped, time‐windowed, and placed on a stable baseline. Only under these conditions can a threshold crossing be meaningfully interpreted as a count assigned to a defined energy bin.

## 6 EVENT COUNTING AND ENERGY BINNING

Once the front end has produced a decision‐ready waveform, the next task is to convert that analog pulse into discrete data. At this stage, photon counting reduces the waveform to two linked decisions: whether a photon interaction has occurred, and, if so, to which energy bin the event should be assigned. These decisions are made by applying threshold‐based logic to a processed waveform whose amplitude, shape, and timing carry information about the deposited photon energy and event timing. That waveform reflects the combined effects of photon interaction physics, carrier transport under the detector operating conditions, weighting‐field geometry determined by the electrode structure, and front‐end pulse processing.

The energy spectrum measured by a PCD is not a direct record of incident photon energies. It is produced by applying threshold‐decision rules to processed analog waveforms that serve as proxies for deposited energy. Energy thresholds therefore do not act on the photons themselves; they act on the waveforms produced by the preceding stages of the measurement chain. Although event counting and energy binning are the first explicitly digital steps in the readout process, their outcomes are determined by the upstream physics of photon interaction, charge transport, pulse formation, and pulse processing.

### 6.1 What constitutes a count

A count is not simply the presence of a pulse. It is the outcome of a decision rule applied to a processed continuous waveform. The readout must therefore extract event‐defining features from the shaped waveform, such as threshold‐crossing time, pulse height, pulse width, or time over threshold, and use them to define discrete digital outcomes.

**FIGURE 10:**
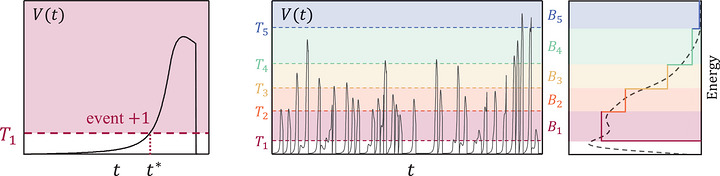
From a processed waveform to a counted event and an energy bin. **Left**: A count is commonly declared at the rising‐edge crossing of the lowest threshold, $T_{1}$. The pulse‐amplitude proxy A (for example, the one defined in Box 5.2) then determines which additional thresholds are exceeded. **Middle**: Multi‐threshold exceedance decisions are converted into bin counts, for example by differencing counts above threshold. This illustrates that the measured count distribution is not a direct record of photon energies, but is instead constructed from threshold decisions applied to a processed waveform, essentially an information compression process (**right panel**).

In many photon‐counting implementations, an event is declared when the processed pulse crosses the lowest threshold on the rising edge of the waveform, and the corresponding crossing time is taken as the event time.^108^ This rule assigns an unambiguous event time and avoids double counting when the same pulse later falls back below the threshold. As illustrated in Figure 10, the event time also affects downstream logic, such as reset‐time enforcement and coincidence arbitration.

Although several event‐defining features can be extracted from the processed waveform, threshold‐based comparator or discriminator logic is favored in PCDs because it can be implemented with the speed and efficiency required for high‐flux operation. One could, in principle, digitize each pulse more completely and use multiple waveform features for event classification, but such approaches are generally more demanding in circuit complexity, timing, power consumption, and pixel area than a small number of threshold decisions. In some PCD readout architectures, event identification may use a more elaborate combination of discriminator outputs, including threshold‐crossing order, threshold‐crossing timing, time‐over‐threshold information, inter‐pixel arbitration, or additional validation logic.^112^, ^113^ Once an event has been identified, additional thresholds can be used to assign the event to an energy bin and thereby encode coarsely sampled spectral information.

### 6.2 How multiple thresholds form energy bins

Once an event has been defined, multi‐threshold discrimination provides a practical route to spectral encoding. Instead of estimating a continuous energy for every photon, the detector asks a sequence of simpler questions: did the processed pulse exceed threshold $T_{1}$? Did it exceed $T_{2}$? Did it exceed $T_{3}$? The set of these exceedance decisions is then converted into bin populations. This architecture is one of the reasons PCDs can provide coarsely compressed spectral information at very high rates.

The conceptual advantage of this approach is simplicity. Energy binning becomes a decision problem rather than a full analog energy measurement. Its limitation is equally important: the quality of the spectral information depends on the stability of the amplitude proxy relative to the threshold set. Any mechanism that shifts the proxy distribution, broadens it, or introduces long tails can change how events are partitioned among bins, even when the post‐object spectrum itself is unchanged.

This also clarifies why the amplitude proxy, for example the one defined in Box 5.2, is so important. Whether that proxy is taken to be the pulse peak height, a threshold‐crossing pattern, or a calibrated time‐over‐threshold quantity, the energy bins are defined in the proxy domain rather than in the underlying photon‐energy domain. The resulting bin populations therefore reflect not only the incident x‐ray spectrum, but also the way in which the detector‐readout chain converts each interaction into a processed waveform.

### 6.3 How thresholds are calibrated

Because thresholds act on an amplitude proxy derived from the processed electrical waveform, the correspondence between this proxy and photon energy must be calibrated experimentally. In practice, calibration data are acquired using photons of known or well‐characterized energies, commonly from radioisotopes or characteristic x‐ray fluorescence lines.^91^, ^114^ For each reference energy, the detector response is measured in the threshold domain by scanning the discriminator threshold and recording the number of counts above threshold. The resulting count‐versus‐threshold curve is cumulative. Its negative derivative with respect to threshold provides an estimate of the differential pulse‐height distribution in threshold space. A representative location of this distribution, such as its peak position, centroid, or another conventionally defined calibration point, is then associated with the known photon energy. Repeating this procedure for multiple reference energies establishes an empirical mapping between photon energy and discriminator threshold.

A clear way to frame the calibration problem is that thresholds are implemented in proxy space but interpreted in energy space. Calibration provides the operational link between these two domains. This link is not determined by the electronics alone; it also depends on sensor material properties, charge transport, weighting‐field geometry, pulse processing, and radiation exposure conditions. In a real detector, the calibration is neither perfectly universal nor perfectly fixed. Pixel‐to‐pixel gain variation, temperature dependence, bias‐voltage dependence, and count‐rate dependence can all shift or distort the threshold‐energy mapping. These effects motivate pixel‐wise threshold trimming, periodic recalibration, and, in some applications, in‐situ calibration strategies, especially under high‐flux operation or over extended acquisition periods.

**FIGURE 11:**
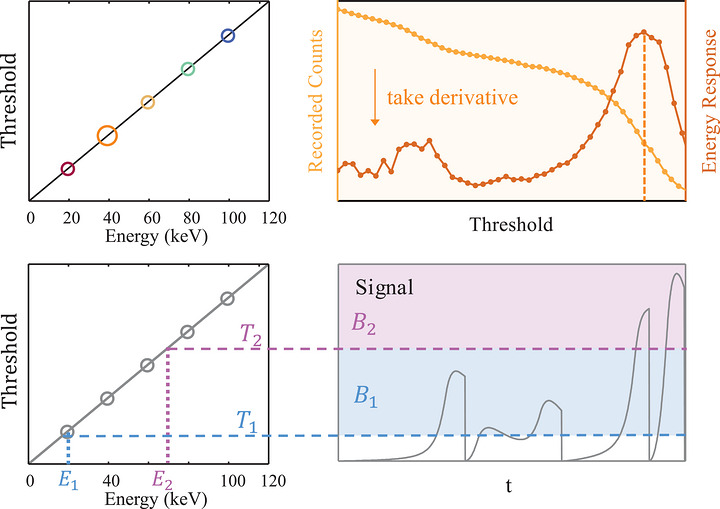
Threshold‐calibration concept, read row by row. **Top row**: calibration is established experimentally. At top right, a threshold scan for a known monoenergetic input produces a counts‐above‐threshold curve; differentiating that cumulative curve yields an estimate of the underlying response distribution in the amplitude‐proxy domain, from which a representative threshold value is assigned to the known energy. Repeating this procedure for several known energies gives the empirical mapping between photon energy and threshold shown at top left. **Bottom row**: the calibrated mapping is then used operationally. At bottom left, desired energy cuts $E_{1}$ and $E_{2}$ are mapped to threshold settings $T_{1}$ and $T_{2}$. At bottom right, those thresholds are applied to the processed waveform to define counted events and assign them to threshold‐defined bins.

Figure 11 summarizes the calibration workflow. Threshold scans acquired at known monoenergetic inputs are used to estimate the corresponding response distributions in threshold space and thereby establish an empirical mapping between photon energy and threshold. That mapping is then used to convert desired energy cuts into implemented thresholds for event detection and spectral binning. The essential point is that this mapping is detector‐ and condition‐dependent rather than universal: any mechanism that shifts, broadens, or distorts the proxy‐domain response will change how photons are distributed among threshold‐defined bins, even when the post‐object spectrum itself remains unchanged.

### 6.4 Why noise still matters at the decision stage

Although electronic noise has already entered through front‐end pulse processing, it remains important at the digital decision stage because thresholds act directly on the processed waveform and its baseline. Baseline fluctuations can generate false triggers if the lowest threshold is set too close to the noise floor. During a true pulse, noise perturbs the apparent threshold‐crossing time and the amplitude proxy used for energy discrimination, thereby introducing timing jitter and bin‐assignment variability. Thus, thresholds do not simply separate signal from noise. They also convert residual analog uncertainty in the processed waveform into uncertainty in digital counting and energy‐bin assignment.

In practice, two competing demands must be balanced. The lowest threshold should be low enough to retain sensitivity to genuine low‐amplitude events, but high enough to keep noise‐induced false counts negligible. Similarly, narrow bin spacing from multiple thresholds improves nominal spectral resolution only when the underlying amplitude‐proxy distribution is stable relative to the threshold separation. Otherwise, electronic noise, baseline fluctuations, and waveform variability increase event migration across bin boundaries and degrade the effective spectral information.

The considerations in this section complete the basic picture of decision logic in PCDs: a processed waveform becomes a count through a threshold‐crossing rule, becomes a spectral measurement through multi‐threshold binning, and becomes interpretable in energy units only through calibration. The next question is why this carefully defined mapping can become distorted, especially at high count rates.

## 7 SPECTRAL DISTORTIONS AND RATE EFFECTS

The threshold logic described in the previous section is best understood in the low‐rate limit, where photon events are well separated in time and rate‐dependent confounding effects are minimal. Under the high‐flux conditions commonly encountered in diagnostic CT, this simple event‐by‐event picture breaks down because count rate becomes part of the measurement process. Waveform overlap, finite decision windows, and flux‐dependent baseline shifts can perturb both event counting and energy‐bin assignment. The measured spectrum is therefore shaped not only by the post‐object photon spectrum and sensor response, but also by the rate‐dependent behavior of the detector–readout chain.

This section examines how these distortions arise and how they appear in measured data. Some distortions originate primarily in the readout electronics, such as dead time, pulse pileup, baseline shifts, and reset behavior. Others originate in photon interaction physics, such as Compton scattering, Rayleigh scattering, and characteristic x‐ray fluorescence emission and escape. Still others arise during charge transport, including charge trapping, detrapping, field‐assisted carrier release, and space‐charge‐induced polarization effects. In practice, the observed distortion often reflects the combined influence of these mechanisms rather than a single isolated effect.

### 7.1 Why high count rate changes the measurement

At high count rates, the detector‐readout chain must evaluate each pulse while the sensor and front‐end electronics may still be recovering from earlier photon interactions. This point is important because a semiconductor detector is not static under irradiation. Its internal physics state can evolve with recent interaction history through residual carrier transport, trapping‐detrapping processes, space‐charge buildup associated polarization effects, front‐end reset, and baseline restoration. The shorter time interval between interactions at high count rate can leave insufficient time for these processes to return to their low‐rate conditions. As a result, the effective meaning of an energy threshold can depend not only on its nominal setting, but also on the local baseline, recent pulse history, and timing constraints imposed by shaping, reset, and recovery logic.

High count rate therefore does more than reduce counting throughput. It can change whether an event is registered, whether it is assigned to the correct energy bin, and whether neighboring events are resolved as separate pulses or merged into a single measured event. Counting performance under CT conditions is therefore inseparable from the rate‐dependent behavior of the detector‐readout chain.

### 7.2 Dead time and pileup

After an event is registered, the front end typically requires a finite time interval before it can reliably process the next event. This interval may be set by shaping dynamics, baseline recovery, explicit reset logic, or additional arbitration in the counting chain. Regardless of the specific implementation, the practical consequence is that events arriving too soon after a previous event may not be registered correctly. Detector dead time provides a simple way to quantify this finite recovery interval and to characterize the resulting rate‐dependent counting limitation.

From the perspective of the electrical waveform, high count rates can cause processed pulses from separate photon interactions to overlap in time. This effect is referred to as pulse pileup. Depending on the relative timing and shape of the overlapping pulses, pileup can produce several distinct outcomes. Two closely spaced events may merge into a single larger pulse and be assigned an artificially high apparent energy. Residual tails from earlier pulses can shift the local baseline and alter the apparent amplitude of later events. In more severe cases, pulse overlap can obscure the required threshold‐crossing pattern and produce count loss.

**FIGURE 12:**
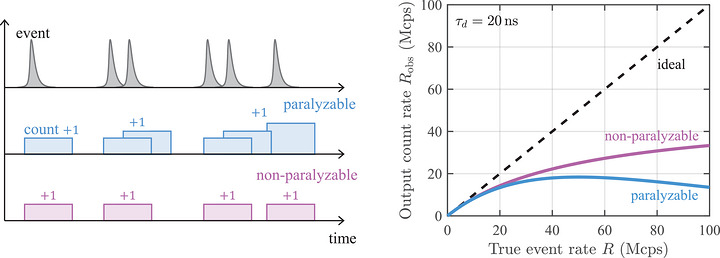
Dead‐time models. **Left**: schematic illustration of paralyzable and nonparalyzable counting. **Right**: observed count rate $R_{\mathrm{obs}}$ versus true count rate R for ideal, nonparalyzable, and paralyzable models with $\tau_{d}=20\;\mathrm{ns}$.

Figure 12 provides a pictorial illustration of dead‐time behavior. In the so‐called paralyzable readout mode, a threshold crossing initiates a signal‐processing time window. If another event occurs during this interval, it can initiate an additional processing window that overlaps with and extends the previous window. As a result, a sequence of closely spaced photon interactions may be recorded as a single event, leading to substantial count loss and unstable counting behavior at high flux. In contrast, in the so‐called non‐paralyzable readout mode, once a processing or reset window has been initiated, additional events arriving during that fixed interval do not initiate a new window. During this interval, at most one count can be recorded, regardless of how many photon interactions occur. The trade‐off is that events arriving within the reset window are not separately counted, but the dead‐time window remains fixed by the readout logic rather than being extended by subsequent events.

To accommodate the high count‐rate requirements of diagnostic CT, practical PCD‐CT readout designs require reset or hold‐off logic that prevents additional events arriving during the active processing window from repeatedly extending that window. The resulting rate response is therefore designed to be non‐paralyzable, or at least approximately non‐paralyzable, rather than paralyzable. Without such logic, events arriving during the recovery interval could continually extend the pulse processing time window, leading to excessive count loss and unstable spectral response at CT‐relevant flux levels. The width of the reset or hold‐off window sets an important lower bound on the effective detector dead time, although the observed rate response can also be influenced by pulse shaping, baseline recovery, pileup, charge transport, and threshold logic.

Box 7.1 summarizes the relationship between the measured mean count rate, the true incident mean count rate, and the effective detector dead time $\tau_{d}$ for PCDs operated in non‐paralyzable and paralyzable readout modes.

**Box 7.1: How do classical dead‐time models limit throughput?**

Two classical first‐order models relate the observed count rate $R_{\mathrm{obs}}$ to the true count rate R through an effective dead time $\tau_{d}$.

For a **nonparalyzable** detector, events arriving during dead time are lost but do not extend the dead time:

$$R_{\mathrm{obs}}=\frac{R}{1+R\tau_{d}}.$$

For a **paralyzable** detector, an event arriving during dead time renews the dead time:

$$R_{\mathrm{obs}}=R\;e^{-R\tau_{d}}.$$

These relations are throughput models rather than full spectral models, but they provide useful first‐order anchors for understanding why count loss becomes substantial as event density increases.

### 7.3 How pulses become misclassified in energy bins

As discussed in Section 6.2, the processed electrical waveform is assigned to energy bins by comparing its amplitude proxy with calibrated thresholds. If the waveform presented to the thresholds changes relative to the calibration condition, the counts recorded in each energy bin can also change. This dependence reflects the full measurement chain. Deposited x‐ray energy determines the initial charge generation, while sensor material properties, operating conditions, and detector geometry influence charge transport. Charge transport determines the induced current. The front‐end electronics then convert this induced current into a processed waveform and an amplitude proxy, and the threshold logic assigns that proxy to an energy bin. Fluctuations or systematic changes at any stage of this chain can therefore cause bin misclassification, or bin migration.

Several physical mechanisms can produce these waveform changes.

#### Charge sharing

Charge sharing can partition the signal from a single interaction across neighboring pixels. If the pixels are processed independently, the result is often multiple reduced apparent amplitudes rather than one correctly assigned event, leading to low‐energy tailing and excess low‐bin counts. Coincidence or charge‐summing schemes can mitigate this effect, although they introduce additional timing complexity and potential throughput penalties. Coincidence‐based schemes are also vulnerable to chance coincidences at high flux.

#### Fluorescence and secondary interactions

Fluorescence and secondary interactions can redistribute deposited energy. A characteristic x‐ray or scattered photon may leave the original interaction site and deposit energy elsewhere, or escape the sensitive sensor volume altogether. The recorded signal may then be spatially distributed across multiple pixels or reduced in apparent amplitude, increasing the probability of bin migration and multi‐pixel ambiguity.

#### Detector pixel geometry

Pixel pitch and detector geometry influence several of these mechanisms simultaneously. Smaller pixels strengthen weighting‐field localization and improve spatial resolution, but they can also increase the probability that a charge cloud with finite lateral extent is shared across neighboring pixels. Larger‐pixel or more planar geometries can reduce multi‐pixel charge partitioning, but they make the measured pulse more sensitive to carrier motion over a larger fraction of the detector thickness. Geometry therefore affects not only spatial resolution, but also the balance between spectral sharpness, depth dependence, and multi‐pixel ambiguity.

#### Charge transport

Charge trapping, detrapping, and polarization effects perturb the charge‐transport process itself. In high‐Z semiconductors such as CdTe and CZT, trapping can reduce the mobile charge contribution and lengthen the waveform timescale. Space‐charge buildup can produce an internal polarization field that modifies the applied electric field, thereby changing carrier transport and the resulting pulse height, width, and timing. These effects can bias the amplitude proxy downward and can enhance ballistic deficit when the pulse becomes slow relative to the shaping and decision windows.

#### Count‐rate dependence

At high count rates, pileup and baseline shifts introduce additional bin‐migration mechanisms. Peak summation can move closely spaced events into artificially high energy bins, whereas residual pulse tails, shortened decision windows, or incomplete recovery can move subsequent events into lower bins or suppress them entirely. Baseline shifts can also change the apparent threshold‐crossing condition. This bidirectional migration is one reason rate‐dependent spectra can be difficult to interpret from the incident post‐object spectrum alone.

**FIGURE 13:**
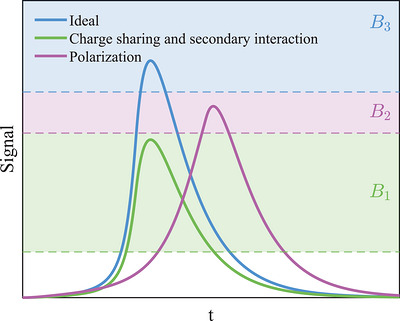
Pulse‐domain origin of bin misclassification. Ideal and distorted shaped pulses are shown with thresholds overlaid. A reduction in pulse amplitude and/or an increase in rise time can change which thresholds are crossed within the decision window, producing bin errors and, in extreme cases, count loss at the lowest threshold.

Figure 13 illustrates a few representative pulse‐domain distortions that can lead to energy‐bin errors. Charge sharing, fluorescence, or other secondary‐interaction processes can reduce the apparent pulse amplitude. Larger‐pixel geometries or degraded charge transport can produce slower pulse rise times and increased depth dependence. Space‐charge buildup can generate an internal polarization field that weakens the applied electric field, thereby changing charge transport and the resulting pulse height, width, and timing. These pulse distortions can alter which thresholds are crossed within the decision window and can therefore bias the estimated post‐object energy spectrum.

These pulse‐domain effects appear in the measured spectrum through several recurring signatures. The first is peak shift, a systematic displacement of the apparent energy scale. In pulse terms, this usually reflects a systematic change in gain or a systematic reduction of the measured amplitude proxy, for example from ballistic deficit or transport degradation. The second is peak broadening, an increase in the spread of recorded responses around the nominal energy value. This often reflects increased variability of the amplitude proxy due to electronic noise, depth dependence, charge sharing, or time‐varying internal fields. The third is low‐energy tailing, which in severe cases can progress to substantial low‐energy redistribution or loss of spectral separation. This signature indicates that events are being redistributed toward lower amplitudes and therefore lower energy bins. Common causes include charge loss, strong ballistic deficit, charge sharing, and pronounced baseline or rate effects.

This pulse‐domain view unifies several PCD performance limitations. Upstream detector physics can shift or broaden the amplitude‐proxy distribution, while rate‐dependent electronics can alter the baseline, timing, and threshold‐crossing behavior. These mechanisms act through the same endpoint: they change the probability that a processed waveform is assigned to one threshold‐defined bin rather than another, thereby biasing the estimated post‐object energy spectrum.

### 7.4 A spectral response view

To characterize energy‐bin misclassification and bin migration, one can introduce the spectral response function (SRF).^91^, ^115^, ^116^ Rather than tracking every pulse in full waveform detail, this approach characterizes the detector by the expected number of counts assigned to each output bin for an incident photon of a given energy and interaction location within the sensor. In its most detailed form, this description retains both spatial and spectral information, giving a spatial‐energy correlation function over a cluster of detector pixels.^117^, ^118^, ^119^ When the response is summed over output pixels, the spatial‐energy correlation function is reduced to a single spectral response function that is convenient for experimental validation,^91^, ^115^ as summarized in Box 7.2.

**Box 7.2: How can spectral distortion be summarized compactly?**

Let $M_{k\ell }$ denote the expected number of counts recorded in bin k per incident photon of energy $E_{\ell }$:

$$M_{k\ell }=E\;\left[\text{counts in bin}\;k|E_{\ell }\right].$$

This matrix defines the spectral response function (SRF),

$$\mathrm{SRF}\left(\text{bin}\;k|E_{\ell }\right)\equiv M_{k\ell }.$$

The matrix M compactly describes how incident photon energy is transformed into recorded bin counts. The sum over k gives the expected count multiplicity produced by one photon of energy $E_{\ell }$. Thus, M captures the combined effects of photon interactions, charge transport, induction geometry, pulse formation, thresholding, and finite‐rate distortion. Changes in transport conditions, electronics settings, or count‐rate environment alter M, and therefore alter the measured spectrum even when the post‐object spectrum itself is unchanged.

The SRF makes explicit that the measured spectrum is a transformed and largely distorted version of the incident post‐object spectrum, and that this transformation depends on detector physics, front‐end electronics, and operating conditions. An important point is that the SRF should not generally be interpreted as a normalized conditional probability. Because one incident photon can produce zero, one, or multiple recorded counts, the sum of the SRF over output energy bins gives the expected count multiplicity for that incident photon energy. This multiplicity can be smaller than, equal to, or larger than one.

This viewpoint is useful because it compresses a complicated detector‐electronics measurement chain into a representation that is both interpretable and operationally useful. Mechanisms such as charge sharing, fluorescence escape, trapping, polarization, dead time, pileup, and thresholding effects then appear as changes in how incident photons are redistributed among recorded bins.

To summarize this section, the preceding subsections show that spectral distortion in PCDs is generally unavoidable under practical operating conditions. This distortion is not a single isolated effect, but the observable consequence of a coupled detector‐electronics measurement chain operating under finite‐rate conditions. The measured spectrum changes whenever this chain shifts relative to calibration or is driven beyond the isolated‐event regime.

## 8 DETECTOR CONTACTS AND DARK CURRENT

PCD pixels are designed to detect femtocoulomb‐scale transient induced‐charge signals at the collection electrodes, as estimated in Box 3.1. In an oversimplified electrostatic picture, the applied bias voltage establishes an electric field in the semiconductor, and any finite conductivity or carrier injection would produce a leakage current superimposed on the radiation‐induced transient signal.^120^, ^121^ If this leakage current were not controlled, it would load the front‐end input, shift the baseline, and compromise the ability of the PCD to resolve single‐photon pulses.

The metal‐semiconductor contacts at the detector electrodes strongly affect carrier injection, leakage current, and baseline stability. Properly designed contacts help suppress unwanted current while allowing efficient collection of radiation‐generated charge. In this paper, we use the term dark current in a PCD‐specific operational sense to refer to currents and charge‐carrier contributions that are not part of the prompt signal from the photon interaction being counted. These contributions can arise from sensor leakage, contact‐related carrier injection, field‐assisted emission, thermal activation, trapping‐detrapping processes, and readout circuitry. This usage should be distinguished from the conventional role of dark current in energy‐integrating detectors, where dark current is mainly associated with the photodiodes and readout electronics that convert scintillation light into an electrical signal, rather than with the scintillation process or optical‐photon transport process. In direct‐conversion PCDs, by contrast, unwanted carriers generated or released during sensor operation can directly influence charge transport and electrical pulse formation, in addition to producing readout‐related baseline loading. These contributions are important in PCD detector physics because they can increase noise, cause baseline drift, and degrade threshold stability.

In this sense, detector contacts are not peripheral details. They are part of the causal measurement chain because they determine whether the sensor can be operated at the high bias needed for rapid charge collection while maintaining a baseline that is sufficiently low and stable for threshold‐based pulse discrimination.

### 8.1 Contacts as boundary control for collection and injection

When a semiconductor sensor is placed between two metal electrodes to form a metal–semiconductor–metal structure, the interfaces do more than provide electrical connection. They set the electrical boundary conditions for charge exchange between the semiconductor and the external circuit, and therefore govern both the undesired injection of carriers from the electrodes and the desired extraction of the radiation‐generated charge that forms the detector signal.

For detector operation, the key question is whether the biased contact pair, under the chosen polarity, can satisfy two requirements at the same time: it must permit efficient collection of the desired radiation‐generated charge, and it must suppress unwanted injection strongly enough that the dark current remains acceptably low. This is the physical logic behind the common use of asymmetric contacts, often arranged so that one contact acts primarily as a blocking contact to suppress carrier injection, while the other acts primarily as a collecting contact for the desired signal charge. Even so, the real behavior of CdTe and CZT contacts is usually more nuanced than the simplified textbook picture because of surface states, surface chemistry, Fermi‐level pinning, bulk defects, and parasitic surface or edge pathways.

This balance becomes especially important in modern PCD‐CT. High‐flux operation pushes the front end toward short pulse‐processing times, often on the order of 10ns and potentially even shorter in future architectures. For a sensor of typical thickness d∼2mm, achieving sufficiently rapid and efficient charge transport on that timescale generally requires substantial bias and therefore strong internal electric field. The same bias that accelerates signal transport, however, can also enhance contact injection, field‐assisted leakage, and parasitic conduction. Contact optimization is therefore not a peripheral materials issue. It is one of the enabling conditions for operating the detector in the high‐field regime required for fast, stable, high‐flux PCD‐CT imaging.

### 8.2 Dark current pathways and their impact on pulse readout

By our definition in the paper, dark current in PCDs is the current that flows through the detector in the absence of radiation (Box 8.1). In a PCD, the requirement is not that this current must vanish, but that it should remain small and stable enough that the shaped baseline stays well separated from the lowest counting threshold. Dark‐current control is therefore a central requirement for reliable pulse counting, low false‐trigger probability, stable energy‐bin assignment, and robust operation at high flux.

Several physical pathways can contribute to the total dark current. Carriers may be injected from the contacts if the relevant interfacial barriers are too low, too thin, or too unstable under bias. Additional carriers may arise through the detector bulk, especially in sensor material containing defects, traps, or field‐activated conduction pathways. Further contributions may travel along surfaces, sidewalls, inter‐pixel gaps, or edge regions, where the field configuration and passivation quality differ from those in the nominal bulk. These pathways respond to different control strategies: contact design acts primarily on injection, crystal quality and resistivity act primarily on bulk leakage, and passivation plus guard structures act primarily on surface and edge leakage.

Dark current affects electrical pulse readout through two coupled mechanisms. First, it loads the front end and shifts the baseline operating point. Second, because it is carried by discrete charges, it produces fluctuations that are filtered by the shaping circuit and appear as additional baseline noise at the thresholding stage (see Box 8.2). The combined effect is a reduced separation between the baseline distribution and the pulse‐amplitude‐proxy distribution, thereby increasing false‐trigger risk and degrading the reliability of energy discrimination.

From a PCD system perspective, the central issue is not the existence of dark current, but whether the resulting baseline shift and baseline fluctuations remain small enough so that stable threshold discrimination can be maintained with acceptably low false‐trigger probability.

### 8.3 Contact optimization as a usable‐bias‐window problem

A physically sound contact design must satisfy two competing requirements. The applied bias must be large enough to establish an internal electric field that supports rapid and efficient charge collection. At the same time, that bias must not drive the detector into a regime in which contact injection, trap‐assisted emission, bulk leakage, or parasitic surface conduction generate enough dark current to compromise threshold stability, count‐rate performance, or spectral fidelity. The practical problem of contact optimization is therefore to maximize the usable bias range over which charge collection is fast while dark‐current‐related degradation remains acceptably small.

For a detector of fixed thickness d, it is often more practical to formulate this principle in terms of the applied bias voltage V rather than the electric field E. Under the common first‐order approximation of a nearly uniform internal field, discussed earlier in Box 3.2, the field strength scales directly with bias voltage and inversely with sensor thickness. This approximation is adequate for design‐level reasoning, although the true internal field may deviate from uniformity because of contact depletion, space charge, polarization, trapped charge, and other nonideal effects.

Within this voltage‐domain viewpoint, the central design objective may be expressed as the existence of a usable bias window,

(10) $$V_{\min,\mathrm{coll}}<V<V_{\max,\mathrm{dark}}.$$

Here $V_{\min,\mathrm{coll}}$ denotes the minimum bias at which charge collection becomes sufficiently rapid and efficient for the intended detector thickness and pulse‐processing time, whereas $V_{\max,\mathrm{dark}}$ denotes the maximum bias at which dark‐current‐induced baseline loading and baseline fluctuations remain small enough for stable threshold discrimination. A favorable contact design is therefore one that makes this interval as wide as possible.

This contact design is especially important for modern PCD‐CT. Short pulse‐processing time pushes the detector toward stronger field and therefore higher bias, particularly for a sensor thickness around 2mm. At the same time, increasing bias can enhance contact injection and other leakage pathways. The scientific value of contact engineering is therefore not merely that it lowers dark current at some nominal operating point. Rather, it expands the high‐bias regime that remains practically usable for rapid, stable, and low‐threshold photon counting. In this sense, contact optimization is what makes the desired high‐field operating regime physically accessible.

**Box 8.1: What controls dark current?**

At the detector level, dark current is best viewed as an operating‐state quantity rather than a fixed material constant:

$$I_{\mathrm{dark}}\equiv I_{\mathrm{dark}}\left(V,T,\phi_{B},\rho ,A_{\mathrm{pix}},d,\text{…}\right),$$

where V is the applied bias, T is temperature, $\phi_{B}$ is an effective contact‐injection barrier, ρ is the effective resistivity of the dominant leakage path, and $A_{\mathrm{pix}}$ is the detector pixel area and d denotes the sensor thickness.

Two limiting regimes are especially useful. If leakage is injection‐dominated, dark current depends mainly on the contact/interface barrier, temperature, and electric field, and may be represented in an effective form as

$$I_{\mathrm{inj}}\left(V,T,\phi_{B}\right)\approx I_{0}\left(T,\phi_{B}\right)\exp\left(\gamma \sqrt{V}\right).$$

If leakage is conduction‐dominated, the dominant dependence shifts toward resistivity, geometry, and bias, leading to an estimate of the form

$$I_{\mathrm{cond}}\left(V,\rho ,A,d\right)\approx \frac{A_{\mathrm{pix}}}{\rho }\frac{V}{d}.$$

The practical message is that contact optimization changes dark current not only by reducing its magnitude, but also by changing how sensitively it responds to bias, temperature, and interface conditions over the intended operating range.

As discussed earlier in Box 3.1, a single diagnostic‐energy x‐ray interaction produces only a femtocoulomb‐scale charge packet. That signal scale provides a useful reference for judging how much dark charge may accumulate during the short interval over which the front end must discriminate photon‐induced pulses. As the numerical estimates below (Box 8.2) make clear, even apparently modest leakage currents can become operationally important once pulse‐processing times are pushed into the ∼10ns regime.

In summary, the central lesson is not merely that dark current should be small. It is that modern PCD‐CT increasingly demands the simultaneous combination of high field, short pulse‐processing time, and low threshold, and that this regime is practically accessible only when the contact system suppresses dark current strongly enough that baseline loading and baseline fluctuations remain compatible with stable threshold discrimination. In this sense, contact engineering is what makes the desired high‐field detector regime usable.

**Box 8.2: When does dark charge become comparable to signal charge?**

As discussed earlier, a single diagnostic‐energy x‐ray interaction generates only a femtocoulomb‐scale charge packet. For example, a representative 60keV event produces

$$Q_{\mathrm{sig}}\left(60\;\mathrm{keV}\right)∼2\text{--}3\;\mathrm{fC},$$

whereas a lower‐energy 20keV event corresponds roughly to

$$Q_{\mathrm{sig}}\left(20\;\mathrm{keV}\right)∼0.7\text{--}1\;\mathrm{fC}.$$

For dark‐current analysis, the relevant additional quantity is the charge accumulated during the effective pulse‐processing window τ:

$$Q_{\mathrm{dark}}\approx I_{\mathrm{dark}}\tau .$$

For a representative short processing time τ=10ns,

1nA→0.01fC,10nA→0.1fC,100nA→1fC.

These values show that leakage in the nanoampere‐to‐tens‐of‐nanoampere range per pixel is not automatically negligible in low‐threshold, short‐window photon counting. In particular, 100nA integrated over 10ns corresponds to about 1fC, already comparable to the signal produced by a low‐energy event near 20keV.

Because dark current is carried by discrete charges, it also exhibits shot‐noise fluctuations. Under a Poisson model, the root‐mean‐square (RMS) dark‐charge fluctuation accumulated over time τ is

$$\delta Q_{\mathrm{dark}}=\sqrt{qI_{\mathrm{dark}}\tau }.$$

Thus, the mean dark‐charge burden scales as $I_{\mathrm{dark}}\tau$, whereas the corresponding RMS fluctuation scales as $\sqrt{I_{\mathrm{dark}}\tau }$. Shorter processing time reduces both, but only if the contact system suppresses leakage strongly enough to preserve a practically usable bias window. Equivalently, stable counting requires sufficient separation between the baseline distribution and the lowest discriminator threshold. From this system‐level viewpoint, the key issue is whether dark‐current‐induced baseline shift and fluctuation leave adequate threshold margin for acceptably low false‐trigger probability.

## 9 THERMAL AND ENVIRONMENTAL CONSTRAINTS IN PCD OPERATION

PCD‐CT places a particularly heavy burden on detector‐side electronics because individual photon events must be processed in real time by dense, high‐bandwidth front‐end circuitry. As a result, thermal management and environmental control are not peripheral implementation details. If temperature is not properly controlled, changes in the thermal operating condition can affect detector performance because charge transport in semiconductor sensors is temperature dependent. Carrier mobility, charge‐cloud diffusion, trapping‐detrapping processes, and thermally activated carrier generation can all vary with temperature. Thermal management and environmental control are therefore part of the physical operating conditions required for a PCD to maintain low noise, stable gain, reliable threshold behavior, and sustained high‐flux performance.

This aspect deserves explicit discussion because it is often underrepresented in broad overviews of PCD‐CT. Much of the literature naturally emphasizes the attractive capabilities enabled by PCD‐CT, including energy‐resolving measurement, improved spatial resolution, dose efficiency, and material‐decomposition capability. By comparison, less attention is often given to the practical operating conditions that must be maintained for these benefits to be realized reproducibly in a clinical environment.

### 9.1 Why thermal burden is a distinctive issue in PCDs

PCD‐CT and EID‐CT differ not only in how the post‐object x‐ray information is encoded into electrical signals for measurements, but also in how intensively the detector‐side electronics must operate. An EID channel generally integrates photocurrent over the exposure‐time window and reports one accumulated quantity per sampling period. A PCD channel, by contrast, must resolve individual charge transients in real time, amplify and shape them, compare them against one or more thresholds, and often maintain local digital state associated with counting, arbitration, coincidence handling, or related event logic. The result is a fundamentally more demanding detector‐side operating regime. Because individual events must be processed one by one and at high speed, PCDs require dense, continuously biased, high‐bandwidth front‐end electronics located close to the pixel array, and the associated power dissipation is converted into heat within the detector‐readout subsystem.

For this reason, self‐heating in a PCD is not a secondary implementation detail. It is a direct consequence of the measurement paradigm itself. In many cases, the dominant detector‐side heat load arises not from x‐ray energy deposited in the sensor, but from the pixel‐level electronics needed to maintain speed, noise performance, and decision fidelity. This observation is especially important in CT, where the detector must operate under high photon flux, rapid readout, and stable calibration over extended acquisition periods. The thermal burden is therefore not incidental to system operation; it is built into the architecture that enables photon counting.

The scientific importance of this thermal burden is that temperature feeds back into the same detector processes that ultimately determine pulse formation, threshold stability, energy‐encoding fidelity, throughput, and long‐term operating stability. Leakage current, carrier mobility, diffusion, trapping and detrapping kinetics, baseline drift, and threshold stability all depend, to varying degrees, on temperature. Thermal behavior is therefore part of the physical operating condition that determines whether the detector remains near a reproducible working point. In this sense, thermal burden is not merely an engineering inconvenience. It is an important demarcating distinction between PCD and EID operation.

### 9.2 Pixel‐level thermal power decomposition

To discuss self‐heating in a physically transparent way, it is useful to decompose detector‐side pixel power into a small number of physically distinct contributions:

(11) $$P_{\mathrm{pix}}\approx P_{A,q}+P_{D}+P_{\mathrm{leak}},$$

where $P_{A,q}$ denotes quiescent analog bias power, $P_{D}$ denotes event/update‐driven switching and processing power, and $P_{\mathrm{leak}}$ denotes leak‐related power associated with dark current under sensor bias.

**Box 9.1: What sets the pixel‐level heat budget?**

A compact detector‐side heat budget for a PCD pixel may be written as

$$P_{\mathrm{pix}}\approx P_{A,q}+P_{D}+P_{\mathrm{leak}},$$

with first‐order backbone scalings

$$\begin{array}{cc} P_{A,q} & \propto \frac{C_{\mathrm{in}}^{2}}{\tau_{s}}\;{\mathrm{ENC}}^{-2},\;P_{D}\approx \alpha \;C_{\mathrm{sw}}\;V_{dd}^{2}\;f,\\ P_{\mathrm{leak}} & \approx I_{\mathrm{dark}}\;V_{b}. \end{array}$$

Here $C_{\mathrm{in}}$ is the effective total input capacitance, $\tau_{s}$ is the characteristic shaping time, ENC is the target equivalent noise charge, α is the activity factor, $C_{\mathrm{sw}}$ is the effective switched capacitance, $V_{dd}$ is the supply voltage, f is the characteristic switching or update rate, $I_{\mathrm{dark}}$ is the dark current, and $V_{b}$ is the sensor bias voltage.

These relations are intended as compact first‐order scaling laws, not exact universal formulas. In particular, $P_{A,q}$ increases when the front end must support larger input capacitance, lower noise, or shorter shaping time. The switched‐capacitance term $P_{D}$ increases with activity and supply swing, but its characteristic rate f should not be interpreted as an arbitrary independent frequency: in a realistic fast PCD front end, it must be chosen consistently with the pulse‐processing timescale and is therefore linked, at the order‐of‐magnitude level, to the timing regime set by $\tau_{s}$. The leak‐related term increases with dark current and sensor bias. Detailed derivations and representative prefactors are provided in Appendix.

This partition separates three different power mechanisms: standing analog bias required to maintain the front end at the desired noise–bandwidth operating point, switched‐capacitance activity associated with local pulse handling and event‐processing functions, and sensor‐side dissipation under high bias in the presence of finite dark current. This distinction is useful because the three terms scale differently with front‐end design, operating speed, bias voltage, temperature, and sensor material. The resulting backbone scalings are summarized in Box 9.1, and their first‐order derivations are given in Appendix.

**Box 9.2: What does a representative PCD‐CT pixel dissipate?**

Using the scaling framework in Box 9.1 and the representative parameters summarized in Appendix E, consider a CdTe/CZT‐like PCD‐CT operating point with

$$p=300\;\mu m,\;d=2\;\mathrm{mm},\;V_{b}=1000\;V,\;V_{dd}=1.2\;V,$$

so that

$$A_{\mathrm{pix}}=p^{2}=0.09\;{\mathrm{mm}}^{2}.$$

For a representative fast front‐end design point with $C_{\mathrm{in}}=200\;\mathrm{fF}$, $\tau_{s}=10\;\mathrm{ns}$, and $\mathrm{ENC}=200\;e^{-}$,

$$P_{A,q}\approx 58\;\mu W.$$

For a representative event/update‐driven switching point with α=0.15, $C_{\mathrm{sw}}=0.7\;\mathrm{pF}$, and f=150MHz,

$$P_{D}\approx 23\;\mu W.$$

For the leak‐related term, using the dark‐current‐density anchors in Appendix E,

$$P_{\mathrm{leak}}∼10\;\mathrm{nW}\;\text{(optimized-contact case)},\;P_{\mathrm{leak}}∼0.1\text{--}1\;\mu W\;\text{(conservative envelope)}.$$

Thus, a representative detector‐side pixel power is

$$P_{\mathrm{pix}}\approx 81\;\mu W,$$

with modest variation depending on the assumed leak‐related operating point. For $A_{\mathrm{pix}}=0.09\;{\mathrm{mm}}^{2}$, this corresponds to

$$\frac{P_{\mathrm{pix}}}{A_{\mathrm{pix}}}\approx 0.9\;\mathrm{mW}/{\mathrm{mm}}^{2}\approx 90\;\mathrm{mW}/{\mathrm{cm}}^{2}.$$

The key point is the hierarchy, not the exact number: for representative fast PCD front ends, quiescent analog bias and event/update‐driven switching are both typically in the tens‐of‐μW‐per‐pixel range and usually dominate the detector‐side heat budget, whereas leak‐related power remains secondary unless dark current becomes substantially elevated.

To anchor these scalings numerically, Box 9.2 presents a representative CdTe/CZT‐like PCD‐CT design point. The goal is not to define a universal pixel power, but to make the expected hierarchy of contributions concrete. The parameter choices and their interpretation are summarized in Appendix E. For a representative fast PCD front end, detector‐side power is typically dominated by quiescent analog bias and event/update‐driven processing, while leak‐related power remains secondary unless dark current becomes substantially elevated. The resulting power density, $∼0.9\;\mathrm{mW}/{\mathrm{mm}}^{2}$ ($=90\;\mathrm{mW}/{\mathrm{cm}}^{2}$), is close to the $∼0.1\;W/{\mathrm{cm}}^{2}$ ($=100\;\mathrm{mW}/{\mathrm{cm}}^{2}$) reference level and well below the $∼1\;W/{\mathrm{cm}}^{2}$ upper range often discussed for highly segmented hybrid pixel detectors. The corresponding per‐pixel power remains of the same broad order of magnitude as published fast hybrid‐pixel front‐end values in the tens‐of‐μW‐per‐pixel range.^108^, ^122^

The hierarchy in Box 9.2 supports an important broader conclusion. In realistic energy‐resolving PCD operation, the dominant detector‐side thermal burden is typically set by the standing analog bias needed to sustain low‐noise, high‐speed front‐end performance together with the additional switched‐capacitance activity required for local event handling. The leak‐related term is more platform dependent and can increase under unfavorable dark‐current conditions, but the existence of substantial detector‐side thermal burden does not rely primarily on leakage alone.

Because a clinical PCD‐CT system contains a very large number of detector elements, the per‐pixel burden has an immediate scanner‐scale consequence. To first order, summing $P_{\mathrm{pix}}$ over all detector elements gives a lower‐bound estimate of the thermal load arising from detector operation alone,

(12) $$\begin{array}{c} P_{\mathrm{scanner}}^{\left(\mathrm{LB}\right)}\approx N_{\mathrm{pix}}P_{\mathrm{pix}}. \end{array}$$

This lower bound does not represent the full scanner thermal budget, since non‐pixel contributions such as peripheral electronics, data transmission, power conversion, and cooling overhead are not included.

A final scope boundary should also be made explicit. The first‐order scalings for $P_{A,q}$ and $P_{D}$ arise from generic front‐end noise‐bandwidth requirements and switched‐capacitance activity, and are therefore more broadly transferable across different PCD sensor technologies. By contrast, the numerical scale of $P_{\mathrm{leak}}$ depends much more strongly on material, contact physics, operating field, and detector architecture. The leak‐related values used in Box 9.2 are intended to represent a CdTe/CZT‐like high‐bias PCD‐CT context. Detector concepts such as deep‐ or edge‐on silicon^9^ may operate under substantially different leakage‐control constraints because absorption length, drift length, junction design, and bias conditions are not the same as in the present CdTe/CZT‐oriented discussion. The broader point of the thermal decomposition is therefore not that all PCD platforms share the same leak‐related burden, but that energy‐resolving operation generically incurs substantial analog and event‐processing thermal cost, while the leakage term remains platform dependent.

Once this pixel‐level framework is established, the next natural question is how the thermal burden of a PCD compares with that of the more familiar EID architecture.

### 9.3 PCD versus EID: A thermal comparison

The same thermal‐budget framework also provides a useful basis for comparing PCDs and EIDs. The distinction is not only the sensor material, but also the temporal mode of operation imposed on the detector‐side electronics. A PCD front end must resolve individual photon‐induced charge transients in real time, typically on nanosecond‐scale or similarly short waveform times. An EID front end, by contrast, integrates detector current over a longer sampling interval and reports one accumulated quantity per readout cycle. The two detector classes therefore operate under different bandwidth and decision‐making requirements.

This difference in characteristic time scale has important thermal implications at the detector front end. For a PCD operating in an event‐resolving mode, the quiescent analog‐bias thermal burden follows the scaling summarized in Box 9.1,

(13) $$P_{A,q,\mathrm{PCD}}\propto \frac{C_{\mathrm{in}}^{2}}{\tau_{s}\;{\mathrm{ENC}}^{2}},$$

where $C_{\mathrm{in}}$ is the effective total input capacitance, $\tau_{s}$ is the shaping time, and ENC is the equivalent noise charge relevant to single‐event discrimination. This term reflects the standing analog cost of maintaining a low‐noise, high‐bandwidth operating point for event‐resolved counting.

An EID channel operating in an integrating mode may be described by a corresponding first‐order scaling

(14) $$P_{A,\mathrm{EID}}\propto \frac{C_{f}^{2}}{t_{\mathrm{int}}\;\sigma_{Q}^{2}},$$

where $C_{f}$ is the integrating or feedback capacitance, $t_{\mathrm{int}}$ is the integration interval, and $\sigma_{Q}$ is the allowed integrated charge‐noise level. Equation (14) is intended only as a first‐order integrating‐readout scaling, not as a universal EID power law. Its value here is conceptual: compared with Equation (13), the longer integration time of an EID tends to relax the bandwidth requirement at the detector front end.

A similar distinction appears in the active processing burden. In PCDs, local activity is tied to rapid pulse handling, threshold evaluation, arbitration, and counting, whereas in EIDs the detector‐side activity is more naturally tied to the cadence of integrated sampling. PCDs may therefore carry an event/update‐driven processing burden that scales with local event activity, whereas EIDs generally do not require the same form of per‐event pulse processing.

This comparison does not imply that every PCD system must dissipate more total detector power than every EID system. Rather, the more important point is that PCDs place tighter demands on detector‐side thermal stability because fast event‐resolved operation, threshold‐based energy encoding, and temperature‐sensitive leakage and baseline behavior directly affect counting fidelity. Thermal control in PCD systems is therefore important not merely for heat removal, but for preserving threshold stability, energy‐encoding fidelity, calibration consistency, and reproducible count‐rate performance.

Once this coupling between detector operation and thermal stability is recognized, the next question is how detector behavior shifts when the operating environment itself changes.

### 9.4 Temperature dependence

As argued above, the key issue in PCD‐CT is not detector‐side power dissipation by itself, but the sensitivity of counting performance to thermal drift. Because detector response is tightly coupled to threshold stability, charge transport, and leakage behavior, temperature control is particularly important. If internally generated heat or external thermal conditions are not managed stably, the detector operating temperature can drift, perturbing several aspects of detector behavior at once. Temperature is therefore not just an environmental variable. It directly affects the operating condition of the detector and thus whether the PCD remains near a reproducible working point.

Three pathways are especially important. First, temperature influences dark current and therefore baseline noise, threshold stability, and low‐energy count fidelity (Section 8). As dark current rises, the associated input current noise generally increases, baseline statistics broaden, and threshold crossings near the lowest bin edge become less stable. Second, temperature can alter carrier mobility, diffusion, and collection time, thereby modifying pulse shape, lateral charge spreading, and timing behavior (Section 3.3). Third, trapping, detrapping, and space‐charge evolution are often thermally activated (Box 8.1), so temperature can affect not only instantaneous signal formation but also the rate at which detector behavior drifts under sustained clinical flux.

From a detector‐characterization perspective, temperature should therefore be viewed as a control variable that can shift charge collection, spectral resolving power, timing resolution, count‐rate tolerance, and long‐term stability simultaneously. It helps determine whether the detector remains near a stable calibration and transport regime or instead drifts away from that regime during operation.

Because of this broad coupling, temperature characterization is most informative when expressed through directly observable detector behavior. Examples include dark current, baseline noise, gain drift, growth of low‐energy tailing, throughput change, and time‐to‐instability under fixed irradiation conditions. Such observables connect thermal control directly to measurable detector performance.

### 9.5 Humidity and surface effects

Humidity enters through a different pathway than temperature. Whereas temperature broadly influences both transport and electronics, humidity acts mainly through surfaces, passivation layers, interfaces, and packaging. Its most direct consequences therefore appear as changes in surface leakage, baseline drift, spatial nonuniformity, or unstable pixel‐to‐pixel offsets.

These effects are particularly relevant in pixelated photon‐counting arrays, where even small baseline shifts can perturb threshold‐crossing behavior and produce count differences across channels. In well‐engineered detector and scanner systems, such environmental effects are managed operationally, but they remain physically relevant because inadequate control can still perturb the detector decision chain at the level of observable counts and bin assignments.

For the purposes of this primer, humidity is not framed as a detector‐reliability topic. Rather, it is introduced as an environmental pathway that can affect detector stability through surfaces and interfaces. Its most natural observables are baseline statistics, leakage maps, drift, and spatial nonuniformity under controlled environmental exposure. These effects matter because they can alter the same pulse‐formation and threshold outcomes that ultimately define practical detector performance.

## 10 CHARACTERIZING PHOTON COUNTING DETECTOR PERFORMANCE

In this section, we discuss how PCD performance can be characterized from a detector‐physics and detector‐readout perspective. We organize the characterization around the causal measurement chain

$$\begin{array}{cc} & N_{eh}\to \left\{Q_{0},\;i\left(t\right)\right\}\to \text{front-end processing}\\ & \;\to \text{pulse proxy}\;A\to \text{threshold/bin outcomes}, \end{array}$$

where each stage is governed by distinct physical and electronic processes and therefore involves a different pathway for performance degradation.

An ideal detector‐performance characterization framework would characterize performance at each stage of the causal measurement chain. This is the most direct way to evaluate detector behavior because it connects measured performance metrics to the underlying physical processes that generate the detector signal. In practice, however, such characterization is usually possible only for detector developers and PCD researchers with direct access to raw counts, threshold‐bin data, pulse statistics, or other detector‐domain observables. Most clinical PCD‐CT users do not have access to these data and instead interact mainly with reconstructed PCD‐CT images. Therefore, alternative characterization frameworks are needed to assess detector performance from reconstructed images, which remains an active area of research.

In this section, we emphasize direct detector‐level characterization, but we also discuss how selected detector characteristics may be inferred indirectly from reconstructed CT images under suitable, and often restrictive, acquisition conditions.

### 10.1 Charge collection and effective signal capture

As shown in the measurement chain above, the first question in PCD performance characterization is how efficiently a PCD converts charge generated by an x‐ray interaction into a usable electrical signal. The answer is more subtle than it may first appear. The detector does not directly report whether every carrier reaches an electrode, nor does it directly measure the total induced charge in an idealized infinite‐time sense. Instead, counting and energy binning are based on a processed waveform produced by a finite‐time readout chain that includes amplification, shaping, discrimination, and reset. What matters in practice is therefore not only the amount of charge generated by the interaction, but also whether the induced signal is delivered with the waveform shape, amplitude, and timing needed to form a reliable pulse‐height proxy for thresholding and bin assignment. Equally important, when no true photon event is present, the background electrical signal must remain sufficiently controlled to avoid spurious counts.

From this perspective, charge collection in a PCD should be understood operationally rather than purely microscopically. Two distinct physical pathways can degrade the usable signal. The first is charge‐carrier transport. Trapping or recombination can reduce the mobile charge available to induce signal, while thermally activated carriers, field‐assisted emission, or carrier injection can produce background signal unrelated to x‐ray interactions. The second is finite‐time electronic signal capture. Because the readout chain observes the induced current over a limited time window, slowly induced current components may arrive too late to be fully represented in the processed waveform. These two pathways are physically different, but their practical consequence is similar: both introduce errors in the effective signal delivered to the downstream decision chain.

Errors in effective signal capture propagate directly into the detector output. They can shift and broaden the pulse‐height‐proxy distribution used for thresholding. At the detector‐output level, this may appear as missed counts near the lowest threshold, migration of events into either lower or higher energy bins, or increased sensitivity to shaping and reset conditions. In contrast, if background fluctuations become too large, they can erode the separation between baseline and true event pulses and increase the risk of false triggers and spurious counts.

This operational viewpoint naturally leads to the next question: once a usable pulse has been formed, how faithfully does that pulse preserve the spectral information carried by the deposited photon energy?

### 10.2 Spectral response function

As discussed in previous sections, each photon interaction in a PCD generates an electrical signal that passes through the detector readout chain, and the event is assigned to a threshold‐defined bin according to the processed signal presented to the comparator. The recorded threshold‐bin counts therefore form a compressed, and potentially distorted, surrogate of the post‐object x‐ray spectrum. Unlike some other photon‐counting applications, such as nuclear medicine measurements that may emphasize the width of a nominally monoenergetic photopeak, the central practical issue in PCD‐CT is not simply how narrow a monoenergetic peak appears. Rather, it is how faithfully deposited photon energy is carried through the full causal measurement chain and encoded in the final threshold‐bin counts.

This distinction matters because the detector output in PCD‐CT is inherently threshold‐based. If events with different deposited energies produce strongly overlapping processed‐signal distributions, thresholding may not separate them reliably. Conversely, when the detector response changes monotonically and predictably with deposited energy, and when the associated fluctuations and distortions remain sufficiently small, neighboring energy ranges can be distinguished more cleanly and with greater sensitivity. Therefore, the relevant performance concept for PCD‐CT is the spectral fidelity of the threshold‐decision process.

For monoenergetic irradiation, detector performance is often summarized by an energy‐resolution metric, commonly expressed through the full width at half maximum (FWHM) of the inferred energy distribution, or equivalently by the resolving power $E/\Delta E_{\mathrm{FWHM}}$. Such measures are useful for describing the width of a monoenergetic response under controlled conditions. However, for a threshold‐based detector in CT, they provide only a partial description. A single width metric does not fully represent threshold ambiguity, bin migration, asymmetric tailing, peak shift, or rate‐dependent distortion under realistic operating conditions.

The most direct consequence of limited spectral fidelity is bin migration near thresholds. When the processed signal corresponding to a fixed deposited energy fluctuates around a threshold, nominally identical events may be recorded in different adjacent bins. Any mechanism that broadens, shifts, or distorts the detector response increases this ambiguity. Modest transport nonuniformity or electronic noise may primarily broaden the response, whereas stronger field distortion, charge sharing, charge loss, fluorescence escape, or pulse pileup may additionally introduce asymmetric low‐energy tails, peak displacement, or more complicated redistribution patterns. Under such conditions, spectral behavior is no longer captured adequately by a single width parameter.

This limitation is especially important in CT, where the detector operates under both high photon fluence rate and a broad polychromatic x‐ray spectrum. High fluence rate increases the probability of pileup and related rate‐dependent effects, so the recorded signal may no longer represent an isolated photon interaction. At the same time, the post‐object spectrum is continuous rather than monoenergetic, so detector performance must be understood in terms of how photons over a range of energies are redistributed into the recorded threshold bins. For this reason, the conventional monoenergetic metric $\Delta E_{\mathrm{FWHM}}$ or $E/\Delta E_{\mathrm{FWHM}}$ is not sufficient for characterizing spectral performance in CT.

A more complete description is provided by SRF, which characterizes how incident or deposited photon energies are redistributed into recorded threshold‐bin outcomes,^91^, ^114^ as illustrated in Box 7.2. Unlike a single‐number energy‐resolution metric, the SRF captures broadening, asymmetry, tailing, bin migration, and count redistribution within one unified framework. From an imaging‐physics perspective, this SRF‐based characterization metric is more closely aligned with the actual role of the detector in PCD‐CT: the detector is not used primarily as an isolated spectrometer, but as a front‐end measurement system that converts the post‐object x‐ray field into multi‐bin count data for downstream CT tasks such as material decomposition, virtual monoenergetic image formation, and quantitative spectral analysis. Any degradation of spectral fidelity can therefore propagate directly into decomposition bias, spectral inconsistency, and reduced quantitative reliability in reconstructed CT images.

Once spectral fidelity is defined at the single‐event level, the next question is how well that fidelity is preserved when many events arrive within comparable processing times.

### 10.3 Count‐rate performance

At low flux, a PCD can often be understood in terms of isolated pulses. At clinical CT flux, however, the detector must process a temporally dense stream of events, so multiple events may compete for the same analog and digital resources in the readout circuitry. Count‐rate performance therefore describes how well the detector maintains reliable event detection and bin assignment as pulses begin to overlap in time.

Several related concepts arise in this regime, including occupancy, dead time, and pileup. A pixel is effectively occupied while a pulse is being formed, processed, evaluated, and returned to a state in which a subsequent event can again be handled reliably. If another photon arrives during this interval, the later event may be missed, merged with the first event, or measured on a distorted baseline. Count‐rate limitation is therefore not merely a counting problem; it is also a spectral‐fidelity problem and, in some detector architectures, a timing problem.

The term **dead time** can be defined at several levels. One may refer separately to shaping time, discriminator response time, reset time, arbitration delay, or other architecture‐specific recovery intervals. These definitions are useful for circuit design because each identifies a specific contribution to count‐rate limitation. For detector performance characterization, however, the more useful quantity is often an **effective dead time** inferred from the relationship between the true event‐arrival rate and the observed count rate. This effective dead time should be understood as a system‐level descriptor rather than a single microscopic time constant, because it can depend on pulse shape, threshold setting, event energy, pixel architecture, and readout logic.

For many PCDs developed for CT, a practical first‐order description of count‐rate response is given by the nonparalyzable model (Figure 12 and Box 7.1),

(15) $$R_{\mathrm{out}}=\frac{R}{1+R\tau_{d}},$$

where R is the true event‐arrival rate at the detector, $R_{\mathrm{out}}$ is the observed output count rate, and $\tau_{d}$ is the corresponding effective dead time. The value of this model is operational: it provides a compact relationship between the reported count rate and the underlying event‐arrival rate. In practice, $\tau_{d}$ is often estimated by measuring $R_{\mathrm{out}}$ over a range of exposure settings and fitting the resulting response curve, using mAs, tube current, or another quantity proportional to the input flux as a surrogate for R.

Within the broader framework of this primer, pileup can be viewed as the high‐flux extension of the same causal measurement‐chain logic developed earlier. Once two or more events overlap within comparable processing times, the detector no longer handles them as independent signal packets. Their waveforms can interfere, leading to count loss, pulse summation, distorted amplitudes, altered bin probabilities, and, in some cases, increased timing ambiguity. Count‐rate performance is therefore the natural context in which to discuss pulse overlap and pileup explicitly, whereas earlier subsections need refer to them only as downstream distortion mechanisms.

The next question is broader than instantaneous throughput: even if the detector performs well initially at a given flux, does it remain in the same operating regime under sustained clinical irradiation?

### 10.4 Stability at clinical flux

The performance dimensions introduced above are often discussed as if they were stationary detector attributes. In practice, however, the performance of a PCD operating at clinical flux may evolve over time. Carrier trapping, detrapping, space‐charge accumulation, and interface or contact changes can gradually alter the internal electric field and, through it, the full signal‐formation chain. Stability at clinical flux should therefore be understood as the ability of the detector to remain in the same operating regime under sustained x‐ray irradiation in clinical practice.

Regular evaluation of PCD performance over time is important because not all degradation is merely incremental. A detector may exhibit mild gain drift or modest spectral broadening while remaining within essentially the same transport regime. However, as trapped charge accumulates, the internal field may become sufficiently distorted that a local low‐field region develops. In the limiting case, the field generated by the accumulated trapped charge can locally offset, or even nearly cancel, the externally applied drift field. Once this occurs, carrier motion slows sharply in that region, induced‐current waveforms become more tailed, and finite shaping and reset windows become increasingly important determinants of the measured signal.

The practical significance of this phenomenon is that several observables may then change together. What may appear experimentally as separate degradations in pulse height, low‐energy tailing, throughput, or coincidence behavior may in fact reflect a single underlying transition in the charge‐transport environment. Accordingly, stability monitoring should not be treated as just one more independent metric beside the earlier ones. Rather, it addresses whether those earlier metrics remain stationary and mutually consistent during sustained operation.

The location of this stability boundary depends on sensor material, sensor fabrication quality, bias conditions, and pixel design. Material permittivity influences how strongly a given trapped‐charge density perturbs the internal field. Applied bias sets the baseline drift field against which trapped charge acts. Pixel geometry and weighting‐field localization influence how strongly delayed carrier motion appears in the measured signal, while shaping and reset choices determine how much slow transport can be tolerated before it becomes effective charge loss or a substantial throughput penalty. These factors do not define separate instabilities; rather, they shift the same underlying boundary between stable operation and field‐distorted operation.

Experimentally, stability is most usefully characterized through a compact set of observables tracked versus time or dose history under fixed flux. Examples include gain drift, low‐energy tail fraction, throughput change, and related event‐distribution metrics. Such observables do not merely indicate that the detector is drifting; they help reveal whether the detector‐readout chain is crossing into a different transport regime.

Before turning to image‐based inference of detector behavior, one additional detector‐level capability deserves brief discussion: the ability to assign an event time from the processed waveform.

### 10.5 Timing resolution and emerging timing capabilities

In a thresholded PCD, the processed detector waveform can in principle support more than one type of measurement. In current implementations, that waveform is used primarily to generate an energy‐related decision variable for thresholding and bin assignment. More generally, however, the same waveform may also contain information about event time, pulse duration, pulse shape, and other features that could become useful in future detector architectures. From this broader perspective, timing resolution is one example of a more general principle: the detector waveform is a rich intermediate signal, and different readout strategies may extract different kinds of information from it.

For timing applications, the recorded event time in a thresholded PCD is usually defined not by the physical instant of x‐ray interaction itself, but by the moment at which the processed waveform crosses a discriminator threshold. Timing resolution is therefore a detector‐system property that emerges from charge induction, signal formation, analog waveform processing, threshold discrimination, and electronic noise.^123^, ^124^ Although timing resolution is not yet a routine performance metric for current clinical PCD‐CT systems, it remains an important physical attribute of PCD operation and could become increasingly relevant if future CT architectures move toward explicit time stamping of individual photon events.^122^, ^125^, ^126^

One possible extension beyond the current use of the processed waveform would be to record the leading‐edge crossing time at the lowest energy threshold and use this quantity as a practical proxy for photon arrival time. Such timing information is not part of the standard output of current clinical PCD‐CT systems, but it represents a physically plausible future capability. If sufficiently accurate time stamps could be obtained for both photon generation at the source and photon detection in a PCD, they could support timing‐aware measurement strategies, including time‐of‐flight‐like concepts^127^, ^128^ or other forms of temporal discrimination in CT. Such approaches remain technically challenging because the required timing precision must be achieved within the high‐flux, energy‐discriminating readout environment of clinical CT. An additional challenge in x‐ray CT is that, unlike positron emission tomography (PET), there is no naturally simultaneous pair of photons generated by a single annihilation event. Therefore, any time‐of‐flight‐like x‐ray CT strategy would require an accurate external timing reference for photon generation at the source, together with sufficiently precise timing of photon detection at the detector. Whether such approaches become practically useful will depend on the achievable timing precision, sustainable count‐rate performance, architectural complexity, and the extent to which timing information provides clinically meaningful value beyond conventional count and spectral measurements.

A useful first‐order approximation is that the timing uncertainty is determined by the ratio of voltage noise to waveform slope at the threshold‐crossing point:^89^, ^122^:

(16) $$\sigma_{t}\approx \frac{\sigma_{V}}{{\left.\frac{dV(t)}{dt}\right|}_{V=V_{\mathrm{th}}}},$$

where $\sigma_{V}$ is the root‐mean‐square voltage noise at the discriminator input, $V_{\mathrm{th}}$ is the threshold used for timing, and $\sigma_{t}$ is the corresponding timing uncertainty. This expression makes the governing tradeoff explicit: steeper leading edges improve timing precision, whereas larger baseline noise degrades it.

Charge‐carrier transport affects timing primarily through its influence on pulse shape. Slow carrier motion, broadened induced‐current waveforms, or electric‐field distortion can reduce the leading‐edge slope after front‐end shaping, while electronic noise further increases timing uncertainty. In addition, fixed‐threshold timing is susceptible to time walk, because pulses of different amplitudes cross the same threshold at different times. Thus, even though timing is not yet a dominant target in routine PCD‐CT characterization, it remains an important physical attribute of the detector‐readout chain and may become increasingly relevant if future system designs seek to exploit event timing more explicitly.

### 10.6 Inferring detector behavior from reconstructed CT images

The primary emphasis of this section has been direct detector‐level characterization. In practice, however, access to detector‐domain data is not uniform across user groups. Detector developers and system researchers may have access to raw counts, threshold‐bin data, count‐rate curves, or other internal detector outputs, whereas clinical medical physicists almost exclusively interact with reconstructed CT images. For this reason, it is useful to recognize that some aspects of detector behavior can, under suitable conditions, also be inferred indirectly from reconstructed PCD‐CT images.

This image‐based route is possible because detector behavior leaves fingerprints in the measured projection data, and those fingerprints can propagate through the reconstruction process into observable image features. Reduced effective signal capture, spectral misregistration, count‐rate loss, and detector instability may all leave signatures in reconstructed CT numbers, image nonuniformity, spectral decomposition behavior, or task‐based image quality. In this sense, reconstructed CT images can sometimes be used not only to assess overall system performance, but also to probe selected aspects of the underlying detector behavior.

At the same time, such inference is necessarily indirect and must be interpreted with care. Reconstructed PCD‐CT images reflect not only detector behavior, but also acquisition geometry, focal‐spot blur, view sampling, patient or object motion, reconstruction kernel, iterative regularization, and other scanner‐level influences. Accordingly, detector‐level inference from images is most convincing when the acquisition mode is well controlled, the reconstruction pipeline is well understood, and the detector‐related fingerprint can be separated, as much as possible, from competing system effects.

A substantial body of work has already been devoted to system‐level assessment of PCD‐CT, including prototype performance studies, technical evaluation of clinical systems, task‐based image‐quality analysis, and investigations of clinically relevant effects such as pileup and detectability. These studies provide valuable insight into the overall performance of PCD‐CT systems, even when they do not isolate intrinsic detector behavior directly.^129^, ^130^, ^131^, ^132^, ^133^ Under more restrictive but practically relevant operating conditions, recent work has further shown that certain detector‐related quantities may be inferred from reconstructed CT images, including effective detector spatial resolution, aspects of spectral‐response behavior, and effective dead‐time behavior.^116^, ^134^, ^135^, ^136^ These image‐based approaches do not replace direct detector characterization. Rather, they provide a practical bridge for CT users who do not have access to raw detector data, while also helping connect detector‐domain physics to image‐domain consequences.

In summary, the characterization dimensions discussed in this section provide a compact conceptual framework for understanding photon‐counting detector performance. Charge collection determines whether deposited energy is converted into a usable pulse. Spectral fidelity determines how faithfully that pulse is represented in threshold‐bin data. Count‐rate performance determines how reliably events can be processed at clinical flux. Stability at clinical flux determines whether these properties remain sufficiently stationary during sustained irradiation. Although the main perspective of this section has been detector centered, the same detector behaviors can, under suitable conditions, leave observable signatures in reconstructed CT images. In this sense, PCD performance is best understood not as a single detector attribute, but as the integrity of the full detector causal measurement chain and its consequences for CT operation.

## 11 DETECTOR‐SIDE DESIGN IN PHOTON‐COUNTING DETECTORS: A PHYSICS SYNTHESIS

Throughout this primer, we have discussed how x‐ray energy deposited in the sensor creates electron–hole pairs at the primary interaction site, and how secondary interactions associated with Compton scattering, Rayleigh scattering, and fluorescence escape can redistribute energy deposition and produce erroneous counts in neighboring pixels. These effects contribute to charge sharing in addition to the broadening caused by thermal diffusion and Coulomb self‐repulsion during charge transport. We have also examined charge transport, signal formation, dark current generation, detector characterization, and the role of front‐end electronics in transforming induced signals into stable digital counts. With these ingredients now in place, it is useful to step back and synthesize them into a single detector‐design picture. This synthesis also returns to the causal measurement‐chain perspective introduced earlier in this primer. Once the detector is viewed as a chain linking energy deposition to energy‐binned digital counts, it becomes clear that absorption, charge transport, leakage control, and readout stability are not separate considerations, but coupled constraints acting on different stages of the same measurement process. In this section, we synthesize the key physics developed throughout this primer to make the competing design requirements explicit, and clarify how absorption, transport, leakage control, and readout considerations become intertwined in practical PCD design.

### 11.1 Absorption sets the thickness requirement

As illustrated in Section 3.1, for normally incident monoenergetic photons, sensor absorption efficiency is given by

(17) $$\eta_{\mathrm{abs}}\left(d\right)=1-\exp\left(-\mu_{\mathrm{att}}d\right),$$

where $\mu_{\mathrm{att}}$ is the linear attenuation coefficient of the sensor material at the photon energy of interest. This relation makes the first detector‐side requirement explicit: if a material has weaker attenuation, a larger thickness is required to achieve the same absorption efficiency, which is directly tied to radiation‐dose efficiency. This is the fundamental reason why high‐Z, high‐density materials such as CdTe, CZT, and GaAs are attractive for diagnostic x‐ray detection: they can achieve high absorption efficiency with comparatively modest thickness. By contrast, materials with weaker attenuation such as silicon generally require greater thickness, alternative detector geometries, or acceptance of reduced dose efficiency. In this sense, attenuation physics sets a thickness requirement before transport and readout considerations even enter. Beyond total absorption efficiency, as discussed in Section 3.1, the relative importance of Compton and Rayleigh scattering also matters because it influences the likelihood of generating erroneous counts in neighboring pixels.

### 11.2 Charge transport and signal formation set the field requirement

Charge transport and signal formation convert the thickness requirement into an electric‐field requirement. As discussed earlier in Section 3.3 and Box 3.2, the characteristic charge‐collection time increases with drift distance and decreases with stronger electric field and more favorable carrier transport properties. These transport scalings lead to a simple practical message: once detector thickness is increased to improve absorption efficiency, higher operating field, higher bias voltage, or better carrier transport properties are generally needed to maintain sufficiently rapid and efficient charge collection, as discussed in Section 4. The same discussion in Section 3.3 also showed that longer transport time increases the opportunity for diffusion‐driven and Coulomb‐driven charge‐cloud expansion, thereby strengthening charge sharing and broadening the formed pulses. The bias voltage, however, cannot be increased without limit. At the material level, the intrinsic breakdown fields of high‐quality semiconductor materials are typically on the order of $10^{5}\;V/\mathrm{cm}$, and are generally much higher than the electric fields used in normal detector operation. Therefore, dielectric breakdown is usually not the first constraint encountered in routine detector operation, although local field enhancement near electrodes, surfaces, or structural nonidealities can still reduce the usable field.

### 11.3 Dark current and leakage mechanisms

The dark current introduces another constraint on PCD design. As discussed earlier in Section 8, the undesirable dark current in a practical detector is not determined by a single material constant alone, but can arise through several coupled physical pathways, including bulk thermal activation of charge carriers, trap‐assisted leakage, charge injection from both electrodes, and surface or edge conduction. In general, a wider band gap helps suppress intrinsic thermal activation of charge carriers, but the observed dark current in real detector‐grade materials is often shaped just as strongly by deep impurity levels, contact barrier properties, interface quality, surface conditions, and other fabrication‐dependent nonidealities. The practical consequence is that the bias increase needed to increase carrier drift speed and improve signal formation can also enhance field‐assisted leakage and worsen baseline drift. As a result, contact must be carefully selected and engineered to suppress dark current such that the detector can be operated under a higher bias voltage to improve energy resolution.^137^

### 11.4 Coupled and competing constraints among thickness, field, and leakage

When these earlier results are assembled into a single detector‐level picture, a fundamental tradeoff in PCD physics becomes clear. Increasing detector thickness improves absorption, but it also lengthens the transport path and tends to slow signal formation. To recover a short collection time, the detector is then pushed toward stronger internal electric field and therefore, for a given thickness, toward a larger bias voltage. Yet stronger field can also increase dark current through contact injection, trap‐assisted emission, and related leakage pathways. The consequence is that absorption, transport speed, and leakage stability cannot be optimized independently. A viable detector is therefore not defined by any one favorable property in isolation, but by whether these coupled constraints can be carefully balanced so as to create a sufficiently broad and usable operating window. This idea is summarized in Box 11.1.

For a given detector thickness, one seeks a bias range that is high enough to support rapid and efficient charge collection, yet low enough that dark‐current‐induced baseline loading and baseline fluctuations remain acceptably small. The scientific value of contact and material optimization is therefore not merely that it reduces leakage at one nominal operating point, but that it enlarges the dynamic range of bias voltages over which rapid, stable, and low‐threshold photon counting remains physically accessible.

**Box 11.1: How can detector‐side design be summarized as a compact operating‐window problem?**

A compact first‐order synthesis is to view detector‐side design through three coupled scalings:

$$\eta_{\mathrm{abs}}\left(d\right)=1-\exp\left(-\mu_{\mathrm{att}}d\right),\;t_{\mathrm{col}}∼\frac{d^{2}}{\mu V},\;I_{\mathrm{dark}}\left(V,d,T\right)\approx I_{0}\;\exp\;\left(\gamma \sqrt{\frac{V}{d}}\right),$$

where $I_{0}$ absorbs the lower‐field dependence on temperature, band gap, barrier properties, trap density, contacts, and surface conditions.

These relations make the central detector‐side competition explicit. First, increasing thickness improves absorption, but also tends to lengthen the collection time. Second, increasing bias helps shorten the collection time, but can also increase dark current through field‐activated leakage.

A viable detector therefore requires a non‐empty operating window in which absorption is sufficient, charge collection is sufficiently fast, and dark current remains tolerable:

$$\eta_{\mathrm{abs}}\left(d\right)\geq \eta_{\min},\;t_{\mathrm{col}}\left(d,\mu ,V\right)\leq t_{\max},\;I_{\mathrm{dark}}\left(V,d,T,\text{…}\right)\leq I_{\max}.$$

Equivalently, for a chosen thickness, the design requirement may be expressed as the existence of a usable bias window, as shown in Equation (10),

$$V_{\min,\mathrm{coll}}<V<V_{\max,\mathrm{dark}},$$

where the lower bound is set by transport requirements and the upper bound by dark‐current‐related degradation. In this sense, detector‐side design is not a one‐dimensional ranking problem, but a constrained optimization in which absorption, transport speed, and leakage control must overlap favorably for the intended application.

### 11.5 Implications for Si, CdTe/CZT, and GaAs

This synthesized physics picture provides a physically meaningful way to understand the potential strengths and limitations of several sensor materials relevant to PCDs, including silicon, CdTe/CZT, and GaAs.

#### Silicon Detectors

Silicon is best understood as an electronically mature material with excellent uniformity, process control, and integration compatibility, but with weaker absorption properties at diagnostic x‐ray energies than the heavier compound semiconductors. From the perspective of Equation (17), this means that silicon generally faces a stronger thickness burden when used for direct‐conversion x‐ray detection. Its smaller band gap also makes intrinsic thermal activation of charge carriers less favorable than in wider‐band‐gap room‐temperature detector materials, although its exceptional material quality and interface control remain major practical advantages.

#### CdTe and CZT Detectors

CdTe and CZT are particularly attractive because they combine high atomic number, high density, and wide band gap, allowing strong stopping power together with room‐temperature operation. Their principal challenges arise not from attenuation physics but from the coupled transport and leakage side of the problem: trapping, polarization‐related field distortion, nonuniformity, and contact‐sensitive dark current remain major practical limitations.

#### GaAs Detectors

GaAs occupies an intermediate region of the design space.^138^, ^139^ It offers stronger x‐ray absorption than silicon together with a wider band gap, and semi‐insulating or compensated GaAs can provide the high resistivity and a potentially low dark current level needed for x‐ray radiation detection. However, achievable material fabrication thickness, material uniformity, and transport performance remain challenges in the practical trade space.

In summary, each material choice represents a different trade‐off among absorption efficiency, charge transport, signal formation, dark current, manufacturability, and operational stability, and therefore occupies a different region of the operating window. This operating window depends not only on the intrinsic material properties but also on the engineering details of the detector implementation. Once a material choice has been incorporated into a clinical PCD‐CT product, the performance of the final system can be assessed quantitatively using metrics such as spatial resolution, noise properties, spectral response, and ultimately radiation‐dose efficiency.

### 11.6 Implications for ASIC design

The synthesis above also clarifies, in retrospect, why ASIC design is not simply an isolated electronics problem. Rather, it must accommodate the signal and noise properties imposed by the sensor and contact physics. Once the sensor material, thickness, and operating bias are chosen, the resulting pulse duration, leakage burden, baseline load, and threshold‐stability requirements are largely set by detector physics. A detector operated at larger thickness and higher bias may achieve adequate absorption and sufficiently rapid charge collection, but it may also impose increased dark‐current stress, greater baseline drift, and broader pulse variability due to transport nonidealities and device nonuniformities. The front end electronics therefore must do more than register threshold crossings. It must shape, stabilize, and process the detector output so that the waveform presented to the comparator remains sufficiently reproducible that a fixed nominal threshold continues to represent the same physical event in a reliable way. In this sense, the detector and ASIC are not separate subsystems joined only at the electrical interface. They are actually two coupled parts of the same measurement chain. The earlier ASIC discussions should therefore be understood as electronic responses to the same detector‐side operating constraints synthesized here.

In summary, detector‐side design in PCDs is governed by a coupled set of constraints rather than by any single material property. Absorption favors sufficient detector thickness, transport favors sufficiently strong electric field for rapid and efficient charge collection, and leakage control limits how aggressively the detector can be biased. The resulting operating window is therefore determined jointly by attenuation, charge transport, and dark‐current behavior. Viewed in this way, the snesor material, bias, transport, leakage, and readout discussions presented throughout this primer are not separate topics, but different aspects of the same coupled detector‐design problem.

## 12 CONCLUSION

This primer has presented a physics‐based framework for understanding how a photon‐counting detector converts post‐object x‐ray photons into threshold‐bin digital data under clinical operating conditions. We organized the discussion around a single causal measurement chain: energy deposition, charge‐carrier creation, transport, signal induction, front‐end waveform formation, threshold decisions, and final bin statistics. From this perspective, photon‐counting CT produces threshold‐defined or bin‐defined measurements through a staged detector–readout process that converts each photon interaction into count data.

This perspective also clarifies how detector behavior should be interpreted and evaluated. Charge sharing, spectral tailing, pulse pileup, baseline shift, threshold instability, and time‐dependent drift should not be viewed as isolated anomalies. Rather, they reflect limitations, distortions, or changing operating conditions at particular links of the same detector–readout chain. Identifying the responsible link, or small set of links, is therefore essential for scientific interpretation, detector evaluation, commissioning, quality control, and future optimization.

A related implication is that PCD performance cannot be captured by any single metric. Meaningful characterization must instead ask a connected set of questions: whether deposited energy is converted into a usable signal, whether that signal is represented faithfully in threshold‐bin data, whether events can still be processed reliably at clinical flux, and whether these properties remain sufficiently stable during sustained operation. Even when detector‐domain observables are not directly accessible, the same underlying behaviors may still leave identifiable signatures in reconstructed CT images under suitable acquisition conditions.

To close this primer, we conclude with a concise physics‐closure checklist.

### 1. Energy deposition and charge creation

Can I explain how an incident x‐ray photon deposits energy in the sensor, how that deposited energy is partitioned between mobile charge creation and other microscopic channels, and how the mean charge yield and its intrinsic fluctuations set the best‐case signal scale?

### 2. Charge transport and signal induction

Can I explain how drift, diffusion, trapping, recombination, weighting‐field geometry, and detector bias jointly determine the induced current waveform, including its depth dependence, charge‐sharing propensity, and the relative roles of electrons and holes?

### 3. Pulse formation and event definition

Can I explain how the induced current is converted by the front‐end electronics into a processed waveform and a discriminator metric, and why the recorded energy bin is determined by threshold decisions applied to a pulse proxy rather than by a direct measurement of photon energy?

### 4. Spectral distortion and high‐rate behavior

Can I explain how noise, threshold uncertainty, charge sharing, coincidence logic, dead time, pileup, and baseline dynamics alter bin assignment, distort the measured spectrum, and generate inter‐bin correlation or count‐rate‐dependent bias?

### 5. Stability under realistic operation

Can I explain how thermal effects, dark current, trapping, space‐charge buildup, contact and surface conditions, and sustained irradiation can shift the detector into a different operating regime, thereby causing gain drift, threshold drift, throughput changes, or time‐dependent spectral distortion?

### 6. CT‐level interpretation

Can I trace an observed detector‐domain or image‐domain signature, such as pulse‐height loss, low‐energy tailing, throughput collapse, spectral inconsistency, or reconstructed‐image nonuniformity, back to the most likely link, or small set of links, in the detector measurement chain?

If these questions can be answered coherently and causally using the physics arguments developed in this primer, then the reader should have a solid foundation for system evaluation, commissioning, quality control, informed interpretation of spectral CT data, and further research innovation in PCD‐CT. Establishing this causal, physics‐based understanding is the central outcome intended by this primer.

## CONFLICT OF INTEREST STATEMENT

Two authors (G.H.C. and R.L.) received research support from GE HealthCare; however, this manuscript does not represent the commercial or scientific views of GE HealthCare.

## Appendix

This appendix summarizes the first‐order derivations underlying the per‐pixel thermal budget introduced in Section 9.2. We use the compact decomposition

$$P_{\mathrm{pix}}\approx P_{A,q}+P_{D}+P_{\mathrm{leak}},$$

where $P_{A,q}$ is the quiescent analog bias power required to maintain the front end at its target noise–bandwidth operating point, $P_{D}$ is the event/update‐driven switching and processing power associated with pixel‐level pulse handling and logic activity, and $P_{\mathrm{leak}}$ is the leak‐related power associated with dark current under sensor bias.

In this first‐order framework, the continuous‐time analog front end is treated as quiescent‐power dominated: the main analog thermal burden is the standing bias required to achieve the desired noise and shaping performance. Additional count‐rate‐dependent activity is grouped into $P_{D}$ through an effective switched‐capacitance model. This choice avoids double counting between standing analog bias and event‐driven processing activity.

## APPENDIX A QUIESCENT ANALOG BIAS POWER IN A PCD FRONT END

Let the effective input capacitance be defined as

(A1)
$$C_{\mathrm{in}}≜C_{\mathrm{sens}}+C_{\mathrm{ext}}.$$

If $\sigma_{v,\mathrm{in}}$ denotes the root‐mean‐square (RMS) input‐referred voltage noise after shaping, then the corresponding input‐referred equivalent noise charge is

(A2)
$${\mathrm{ENC}}_{Q}≜C_{\mathrm{in}}\sigma_{v,\mathrm{in}},\;\mathrm{ENC}≜\frac{{\mathrm{ENC}}_{Q}}{e}.$$

For a thermal‐noise‐dominated MOS input device, the input‐referred voltage‐noise power spectral density scales as^140^, ^141^

(A3)
$$S_{v,\mathrm{in}}∼\frac{k_{B}T}{g_{m}},$$

where $g_{m}$ denotes the transconductance. After shaping with characteristic time $\tau_{s}$, integrating this spectral density over the effective noise bandwidth, which scales as $1/\tau_{s}$, gives

(A4)
$$\sigma_{v,\mathrm{in}}^{2}\approx \frac{K_{n}k_{B}T}{g_{m}\;\tau_{s}},$$

where $K_{n}$ absorbs shaper‐dependent constants. It then follows that

(A5)
$${\mathrm{ENC}}_{Q}^{2}\approx K_{n}k_{B}T\;\frac{C_{\mathrm{in}}^{2}}{g_{m}\;\tau_{s}},$$

which can be rearranged to yield

(A6)
$$g_{m}\approx K_{n}k_{B}T\;\frac{C_{\mathrm{in}}^{2}}{\tau_{s}\;{\mathrm{ENC}}_{Q}^{2}}.$$

Using the strong‐inversion relation $g_{m}\approx 2I_{D}/V_{\mathrm{ov}}$, where $V_{\mathrm{ov}}$ denotes the MOS overdrive voltage, the corresponding quiescent analog bias power scales as^140^, ^141^

(A7)
$$P_{A,q}\approx \xi V_{dd}I_{D}\approx \xi V_{dd}\;\frac{V_{\mathrm{ov}}}{2}\;K_{n}k_{B}T\;\frac{C_{\mathrm{in}}^{2}}{\tau_{s}\;{\mathrm{ENC}}_{Q}^{2}},$$

where ξ is an overhead factor that accounts for bias‐current usage beyond the idealized input device alone. Equivalently,

(A8)
$$P_{A,q}\propto \frac{C_{\mathrm{in}}^{2}}{\tau_{s}\;{\mathrm{ENC}}^{2}}.$$

This is the first‐order scaling used in the main text to describe the standing analog burden: quiescent analog bias power increases when the input capacitance is larger, the shaping time is shorter, or the target ENC is more stringent. In many continuous‐time PCD front ends, this standing bias is a dominant contributor to the analog thermal budget.

## APPENDIX B ANALOG BIAS POWER IN AN INTEGRATING EID CHANNEL

For comparison with Section 9.3, consider an energy‐integrating detector (EID) channel operating over an integration time $t_{\mathrm{int}}$. If $\sigma_{Q}$ is the allowed RMS uncertainty in the integrated charge measurement and $C_{f}$ is the effective integrating capacitance, then

(B1)
$$\sigma_{V}∼\frac{\sigma_{Q}}{C_{f}}.$$

Using

(B2)
$$S_{v}∼\frac{k_{B}T}{g_{m}},\;B_{\mathrm{eff}}∼\frac{1}{t_{\mathrm{int}}},$$

gives

(B3)
$$\sigma_{V}^{2}∼\frac{k_{B}T}{g_{m}\;t_{\mathrm{int}}},$$

and therefore

(B4)
$$g_{m}\propto \frac{k_{B}T\;C_{f}^{2}}{t_{\mathrm{int}}\;\sigma_{Q}^{2}},\;P_{A,\mathrm{EID}}\propto \frac{C_{f}^{2}}{t_{\mathrm{int}}\;\sigma_{Q}^{2}}.$$

The main value of this scaling is comparative: the longer integration time in an EID channel relaxes the analog bandwidth requirement and therefore the quiescent analog‐bias burden.

## APPENDIX C EVENT‐DRIVEN SWITCHING POWER AND LEAK‐RELATED POWER

To first order, event/update‐driven switching power follows the standard switched‐capacitance scaling^142^, ^143^

(C1)
$$P_{D}\approx \alpha C_{\mathrm{sw}}V_{dd}^{2}f,$$

where $C_{\mathrm{sw}}$ is the effective switched capacitance, α is the activity factor, and f is the characteristic switching or update rate. In the present appendix, $P_{D}$ is defined broadly enough to include pixel‐level event‐processing activity associated with threshold handling, arbitration, counting, and other switched‐node operations that scale with pulse processing rate. Although Equation (C1) is commonly introduced as a CMOS digital‐power law, its role here is more general: it is used as a compact first‐order model for the event‐driven switched‐capacitance burden of the readout chain.

The leak‐related contribution arises from dark current under sensor bias:

(C2)
$$P_{\mathrm{leak}}\approx I_{\mathrm{dark}}\;V_{b}.$$

Equivalently,

(C3)
$$I_{\mathrm{dark}}=J_{\mathrm{dark}}A_{\mathrm{pix}},\;A_{\mathrm{pix}}=p^{2},$$

so geometry and dark‐current density are separated explicitly. Because many leakage pathways are thermally activated, leak‐related power generally increases with temperature through

$$T↑\Rightarrow I_{\mathrm{dark}}↑\Rightarrow P_{\mathrm{leak}}↑.$$

## APPENDIX D SUMMARY FORM

Combining the first‐order component models gives

(D1)
$$P_{\mathrm{pix}}\approx \underset{P_{A,q}}{\underset{︸}{\xi V_{dd}\frac{V_{\mathrm{ov}}}{2}K_{n}k_{B}T\frac{C_{\mathrm{in}}^{2}}{\tau_{s}{\mathrm{ENC}}_{Q}^{2}}}}+\underset{P_{D}}{\underset{︸}{\alpha C_{\mathrm{sw}}V_{dd}^{2}f}}+\underset{P_{\mathrm{leak}}}{\underset{︸}{I_{\mathrm{dark}}V_{b}}}.$$

This expression makes the dominant control knobs explicit: $P_{A,q}$ is governed primarily by the standing noise–speed operating point and $C_{\mathrm{in}}$, $P_{D}$ by event/update‐driven switched‐capacitance activity in the pixel readout chain, and $P_{\mathrm{leak}}$ by dark‐current control under high bias. In this first‐order formulation, no separate event‐driven analog term is introduced, because the analog front end is treated as quiescent‐power dominated and the additional rate‐dependent processing burden is grouped into $P_{D}$ to avoid double counting.

## APPENDIX E REPRESENTATIVE PARAMETER SET USED IN THE NUMERICAL LEAKAGE AND THERMAL ESTIMATES

The numerical estimates in Box 9.2 are intended as order‐of‐magnitude engineering estimates for a realistic PCD‐CT operating point, not as a universal detector specification. Accordingly, Table E1 collects representative detector geometry and bias conditions, leakage‐related quantities used either for a compromised‐blocking stress test or for detector‐side dark‐current power estimates, and front‐end assumptions used to estimate the quiescent analog power $P_{A,q}$, the event/update‐driven digital power $P_{D}$, and the leak‐related power $P_{\mathrm{leak}}$.

**TABLE E1:** Representative parameter set used in the numerical leakage‐current and thermal estimates.

Parameter	Value used	Role
p	300μm	pixel pitch
d	2mm	detector thickness
$V_{b}$	1000V	sensor bias
$V_{dd}$	1.2V	supply voltage
ρ	$10^{9}\text{--}10^{11}\;\Omega \cdot \mathrm{cm}$	effective conductive‐path resistivity
$C_{\mathrm{in}}$	200fF	effective input capacitance
$\tau_{s}$	10ns	shaping time
ENC	$200\;e^{-}$	target noise level
$V_{\mathrm{ov}}$	0.2V	MOS overdrive voltage
ξ	1.5	analog‐bias overhead factor
$K_{n}$	20	effective noise–shaping prefactor
$C_{\mathrm{sw}}$	0.7pF	effective switched capacitance
α	0.15	activity factor
f	150MHz	characteristic switching/update rate
$J_{\mathrm{dark}}$	$40\text{--}300\;\mathrm{pA}/{\mathrm{mm}}^{2}$, $1\text{--}10\;\mathrm{nA}/{\mathrm{mm}}^{2}$	practical dark‐current anchors

The geometric and bias values in Table E1, especially p, d, and $V_{b}$, represent a realistic CdTe/CZT‐like PCD‐CT operating point and are used directly in Box 9.2. Quantities such as $V_{dd}$, $C_{\mathrm{in}}$, $\tau_{s}$, ENC, $V_{\mathrm{ov}}$, and $C_{\mathrm{sw}}$ likewise have direct physical or circuit‐level interpretations, although their exact values remain implementation dependent.

The resistivity range $\rho =10^{9}\text{--}10^{11}\;\Omega \cdot \mathrm{cm}$ is used only in Appendix F, where it represents an illustrative effective conductive‐path resistivity for a compromised‐blocking stress test. It is not assigned to a specific detector material and should not be interpreted as the nominal bulk resistivity of a properly engineered blocking‐contact pixel.

By contrast, the leak‐related power estimates quoted in Box 9.2 are expressed through $J_{\mathrm{dark}}$. The lower range is guided by reported CdTe/CZT dark‐current values under favorable blocking conditions, whereas the higher $1\text{--}10\;\mathrm{nA}/{\mathrm{mm}}^{2}$ range is introduced as a conservative practical envelope for less ideal blocking, surface conduction, field crowding, or elevated‐temperature operation.^144^, ^145^, ^146^

The front‐end quantities in Table E1 do not all play the same role. Parameters such as $C_{\mathrm{in}}$, $\tau_{s}$, ENC, $V_{\mathrm{ov}}$, $C_{\mathrm{sw}}$, and, to a lesser extent, f, may be viewed as representative design‐point quantities for a fast pixel front end. By contrast, α, ξ, and $K_{n}$ are compact first‐order modeling coefficients.

More specifically, α is the average activity factor in the switched‐capacitance estimate $P_{D}\approx \alpha C_{\mathrm{sw}}V_{dd}^{2}f$, so α=0.15 should be interpreted as a moderate representative switching‐activity assumption for pixel‐local event‐processing circuitry. The factor ξ accounts for practical analog‐bias overhead beyond a single idealized transistor, including implementation burden in the CSA, shaper, discriminator, and associated bias circuitry. The prefactor $K_{n}$ absorbs shaper‐dependent and noise‐model‐dependent constants when the detailed analog analysis is reduced to the compact first‐order estimate for $P_{A,q}$. Accordingly, $K_{n}=20$ should be read as a representative effective noise–shaping prefactor for order‐of‐magnitude thermal budgeting, not as a universal shaper constant.

The choice f=150MHz should likewise not be interpreted as a universal detector clock frequency or as a direct surrogate for photon count rate. Rather, it represents a characteristic event/update‐driven switching timescale chosen to be broadly consistent, at the order‐of‐magnitude level, with the assumed nanosecond‐scale pulse‐processing regime, especially $\tau_{s}=10\;\mathrm{ns}$, so that the resulting estimate of $P_{D}$ reflects plausible switched‐capacitance activity associated with threshold handling, arbitration, counting, and related pulse‐processing operations.

## APPENDIX F ILLUSTRATIVE LEAKAGE‐CURRENT AND LEAKAGE‐POWER SCALE FOR A COMPROMISED CONDUCTIVE PATH

As an upper‐envelope stress test, consider an effectively conductive path spanning the full pixel area and detector thickness. For an effective conductive‐path area $A_{\mathrm{eff}}$, effective resistivity ρ, detector thickness d, and applied bias $V_{b}$, the corresponding ohmic leakage‐current estimate is

$$I_{\mathrm{leak}}\approx \frac{A_{\mathrm{eff}}}{\rho }\frac{V_{b}}{d}.$$

For the present per‐pixel estimate, we take $A_{\mathrm{eff}}=A_{\mathrm{pix}}=p^{2}$, so that

(F1)
$$I_{\mathrm{leak}}\approx \frac{A_{\mathrm{pix}}V_{b}}{\rho d},\;A_{\mathrm{pix}}=p^{2}.$$

For a representative PCD‐CT geometry,

$$\begin{array}{cc} p & =300\;\mu m=0.03\;\mathrm{cm},\;d=2\;\mathrm{mm}=0.2\;\mathrm{cm},\\ V_{b} & =1000\;V, \end{array}$$

which gives

$$A_{\mathrm{pix}}=p^{2}=9\times 10^{-4}\;{\mathrm{cm}}^{2}.$$

Equation (F1) then yields

$$I_{\mathrm{leak}}\approx \frac{4.5}{\rho }\;A,\;\left(\rho \;\text{in}\;\Omega \cdot \mathrm{cm}\right).$$

For illustrative effective conductive‐path resistivities

$$\rho =10^{9},\;10^{10},\;10^{11}\;\Omega \cdot \mathrm{cm},$$

the corresponding leakage currents are approximately

4.5nA,0.45nA,45pA.

The associated leakage power is

(F2)
$$P_{\mathrm{leak}}=I_{\mathrm{leak}}V_{b}\approx \frac{A_{\mathrm{pix}}V_{b}^{2}}{\rho d},$$

which gives approximately

4.5μW,0.45μW,45nW,

for the three resistivity values above.

These values are intended only as order‐of‐magnitude conductive‐path stress‐test estimates for compromised blocking, not as predictions for a properly engineered blocking‐contact pixel. Likewise, the chosen ρ values are illustrative effective resistivities of a dominant leakage path rather than nominal bulk resistivities of a specific detector material. Their purpose is to show how quickly leakage‐related current and power can become consequential once blocking is degraded.
